# Directed evolution as an approach to increase fructose utilization in synthetic grape juice by wine yeast AWRI 796

**DOI:** 10.1093/femsyr/foac022

**Published:** 2022-04-26

**Authors:** Michelle E Walker, Tommaso L Watson, Christopher R L Large, Yan Berkovich, Tom A Lang, Maitreya J Dunham, Sean Formby, Vladimir Jiranek

**Affiliations:** Department of Wine Science, The University of Adelaide, PMB 1, Glen Osmond, SA 5064, Australia; Department of Wine Science, The University of Adelaide, PMB 1, Glen Osmond, SA 5064, Australia; Department of Genome Sciences, University of Washington, 3720 15th Ave NE, Seattle, WA 98195, United States; Molecular and Cellular Biology Program, University of Washington, Seattle, WA 98195, United States; Department of Wine Science, The University of Adelaide, PMB 1, Glen Osmond, SA 5064, Australia; Department of Wine Science, The University of Adelaide, PMB 1, Glen Osmond, SA 5064, Australia; Department of Genome Sciences, University of Washington, 3720 15th Ave NE, Seattle, WA 98195, United States; Bioinformatics Graduate Program, University of British Columbia, Genome Sciences Centre, BCCA, 100-570 West 7th Avenue, Vancouver, BC, V5Z 4S6, Canada; Department of Wine Science, The University of Adelaide, PMB 1, Glen Osmond, SA 5064, Australia; Australian Research Council Training Centre for Innovative Wine Production, PMB 1, Glen Osmond, SA 5064, Australia

**Keywords:** fructose, directed evolution, *Saccharomyces cerevisiae*, genomics, fermentation, CRISPR/Cas9

## Abstract

In winemaking, slow or stuck alcoholic fermentation can impact processing efficiency and wine quality. Residual fructose in the later stages of fermentation can leave the wine ‘out of specification’ unless removed, which requires reinoculation or use of a more fructophilic yeast. As such, robust, fermentation efficient strains are still highly desirable to reduce this risk. We report on a combined EMS mutagenesis and Directed Evolution (DE) approach as a ‘proof of concept’ to improve fructose utilization and decrease fermentation duration. One evolved isolate, Tee 9, was evaluated against the parent, AWRI 796 in defined medium (CDGJM) and Semillon juice. Interestingly, Tee 9 exhibited improved fermentation in CDGJM at several nitrogen contents, but not in juice. Genomic comparison between AWRI 796 and Tee 9 identified 371 mutations, but no chromosomal copy number variation. A total of 95 noncoding and 276 coding mutations were identified in 297 genes (180 of which encode proteins with one or more substitutions). Whilst introduction of two of these, *Gid7* (E726K) or *Fba1* (G135S), into AWRI 796 did not lead to the fermentation improvement seen in Tee 9, similar allelic swaps with the other mutations are needed to understand Tee 9’s adaption to CDGJM. Furthermore, the 378 isolates, potentially mutagenized but with the same genetic background, are likely a useful resource for future phenotyping and genome-wide association studies.

## Teaser

Chemical mutagenesis and directed evolution as an approach to improve fructose utilization during wine fermentation.

## Abbreviations

CNV: copy number variant
EMS: ethyl methane sulfonate
TA: titratable acidity
YAN: yeast assimilable nitrogen

## Introduction

*Saccharomyces cerevisiae* is the principal *Saccharomycetes* yeast in both spontaneous and inoculated wine fermentations (Fleet et al. 1984, Heard and Fleet 1985). The interaction between the yeast and grape juice influences not only the wine aroma profile, but whether fermentation progresses quickly, slows down or, in the worst-case scenario, arrests. These sluggish and stuck fermentations are problematic as they can affect production time and increased the risk of wine spoilage and oxidation. Slow/stuck fermentations are typically associated with nutrient limitation, or adverse media composition, but can also reflect the yeast strain's capacity to take up sugars *per se*, or differences between glucose and fructose uptake and utilization (Bisson 1999, Berthels et al. 2004, 2008, Guillaume et al. 2007).

Whilst glucose and fructose are the primary hexose sugars in grape juice (in near equimolar proportions), they are utilized at different rates by the glucophilic *S. cerevisiae* (Guillaume et al. 2007), with the less preferred fructose predominating in the latter stages of fermentation when nutrients are depleted and alcohol is high. The low ratio of glucose to fructose, itself reported to cause stuck fermentations (Schütz and Gafner 1993), can make rectifying a problematic or failed fermentation challenging without reinoculation and/or addition of nutrients.

Hexose sugars are transported into the cell and activated through phosphorylation prior to utilization as carbon and energy sources. Key to the preferential utilization of glucose by *S. cerevisiae* is the substrate affinity of the hexose transporters and sugar phosphorylating enzymes (reviewed in Rodicio and Heinisch 2009). The *HXT* family of transporters responsible for the facilitated diffusion into the cell, have a higher affinity for glucose compared to fructose (Kruckeberg 1996). This affinity is concentration dependent (Boles and Hollenberg 1997), with Hxt1 and Hxt3 considered low-affinity transporters, Hxt4 moderately low-affinity, and Hxt2, Hxt6, and Hxt7 high-affinity. Interestingly, improved fructose utilization is associated with allelic variants of Hxt3 (Guillaume et al. 2007, Zuchowska et al. 2015) as well as a high affinity fructose/H^+^ symporter, Fsy1 (de Sousa et al. 2004, Galeote et al. 2010) found in some wine strains considered fermentation efficient e.g. EC1118 and Fermichamp® (Borneman et al. 2016). Whilst the differential expression of *HXT1-7*, *FSY1*, and *GAL2* (Perez et al. 2005, Nadai et al. 2021) can be correlated with sugar utilization, other important factors include nitrogen utilization and ethanol tolerance, which are also genetically determined (Dequin and Casaregola 2011, Peltier et al. 2019, Kessi-Pérez et al. 2020).

Once taken up, fructose is directly phosphorylated to fructose-6-phosphate, whilst glucose is first phosphorylated to glucose-6-phosphate, before being converted to fructose-6-phosphate by phosphoglucose isomerase (PGI). The initial phosphorylation is undertaken by three sugar kinases, hexokinase isozymes Hxk1and Hxk2, which phosphorylate glucose and fructose, albeit at different rates, and glucokinase, which is specific to glucose (Serrano and Delafuente 1974, Berthels et al. 2004, Guillaume et al. 2007). The differences in substrate affinity (*K_m_* and *V_max_*) for glucose and fructose between these enzymes, together with their differential expression (Rossignol et al. 2003), may also contribute towards the differences in glucose and fructose utilization between wine strains (Berthels et al. 2008). After the isomerization reaction to fructose-6-phosphate, the pathways of metabolism of fructose and glucose do not differ (Boulton et al. 1998). The reaction goes to pyruvate, which then, depending on the conditions, will be metabolized in different ways (Berthels et al. 2008).

Stuck and sluggish fermentation are commonplace and are difficult to avoid, as there can be many reasons for their occurrence, which makes managing such situations difficult requiring both methods of prevention and correction (Bisson and Butzke 2000, Bisson 2005). Grape juice is not a favourable environment for yeast. Juice pH, yeast assimilable nitrogen (YAN) deficiency, the presence of other microorganisms in the initial stages of fermentation, temperature extremes, and many other parameters influence the final outcome (Alexandre and Charpentier 1998). Furthermore, the accumulation of alcohol limits growth, through disruption of the plasma membrane, intracellular organelles, and proteins including those involved in sugar transport (Berthels et al. 2004, Stanley et al. 2010). For this reason, the generation of improved strains, which are better able to utilize fructose under such conditions, and so complete fermentation are highly desirable.

To date, recombinant DNA technology has provided an array of proof-of-concept strains with specific genetic modifications, which are well characterized (reviewed in Pretorius 2000). Whilst ML01 (Husnik et al. 2006) and ECMo01 (Coulon et al. 2006) are approved in the US (Pérez-Torrado et al., 2015), such yeasts are yet to be fully embraced by the Australian and European wine industries because of consumer rejection and statuary legislation. As such, improvement strategies have relied on nonrecombinant techniques, namely clonal selection, mutagenesis, hybridization, and rare mating (Pretorius 2000, Alperstein et al. 2020, Eldarov and Mardanov 2020). With the exception of quantitative trait loci (QTL) assisted breeding (Laffort 2021), these techniques show promise but they do not allow for targeted modifications *per se*, and may compromise other beneficial attributes. Directed Evolution (DE) represents a complementary paradigm in the development of new wine yeasts, which is being explored (McBryde et al. 2006, Kutyna 2008, López-Malo et al. 2015). The method relies on genome plasticity (Marsit and Dequin 2015), enabling the original population to diverge and evolve over successive generations to a particular environment, with the fittest eventually dominating the population.

The aim of this study was to improve fructose utilization in a commercial wine yeast, AWRI 796, with the view of alleviating poor sugar catabolism (namely of fructose) during the later stages of fermentation. The experiment was a ‘proof of concept’ using a combination of random mutagenesis and experimental evolution to obtain a robust strain. The novelty of the design was the use of limited fructose as a sole carbon source in Chemically Defined Grape Juice Medium (CDGJM), which mimics wine must. An evolved strain, Tee 9, was isolated that showed improved fermentative capacity in this medium but not the juices subsequently tested, and as such may be of limited benefit to industry. Nevertheless, the strain is still of interest in relation to yeast biology, and as such the genomes were sequenced in order to identify nonsynonymous single nucleotide polymorphisms (SNPs) in genes related to fermentation. *Gid7* (E726K) and *Fba1* (G135S) variants were evaluated as allelic swaps (mutant for wild type) as a step towards understanding the improved phenotype.

## Materials and methods

### Media

Yeast were routinely maintained on YEPD (1% yeast extract, 2% bacteriological peptone, and 2% D-glucose) solidified with 2% agar (Fink 1970). For YEPF, D-fructose replaced D-glucose. CDGJM (Henschke and Jiranek 1993) was prepared according to (McBryde et al. 2006) except that ammonium sulfate was replaced with a mixture of amino acids and ammonium chloride (Table S1, Supporting Information). The total nitrogen concentration (supplied as a 25x amino acid solution) was 600 mg l^–1^ (551 mg l^–1^ YAN) for the DE experiment and fermentation trials unless specified. D-glucose and D-fructose concentrations varied depending upon the experiment; the variation denoted as a suffix, e.g. CDGJM_4F contained 4 g l^–1^ fructose while CDGJM_G+F50 contain 25 g l^–1^ of each sugar. Solutions were sterilized by filtration (0.22 µm). CDGJM starter medium was supplemented with ergosterol (10 mg l^–1^) and Tween 80^®^ (0.5 ml l^–1^), and the glucose and fructose concentrations halved. Semillon (2016, Coombe Vineyard, University of Adelaide; 233.91 g l^–1^ sugar (as glucose and fructose), 96 mg l^–1^ YAN; TA 4.9 g l^–1^; pH 3.18) juice was defrosted at 2°C and filter sterilized (0.22 μm). Starter medium was an equal volume of YEPD and Semillon juice.

### Yeast strains and maintenance

AWRI 796 (Maurivin, Australia) and Fermichamp® (DSM, Netherlands) were supplied as activated dry wine yeast. Yeast samples were rehydrated in sterile water (20 min), before overnight culturing in YEPD (28°C with shaking) and plating on YEPD agar. Clonal isolates grown in 25 ml YEPD were stored as 1 ml glycerol (26%) stocks at −80°C. A clonal isolate of AWRI 796 (clone 5), provided the genetic background for the DE experiment. Fermichamp® was used as a reference because of its high affinity for fructose. Genetic diversity of the starting population was increased by chemical mutagenesis using ethyl methyl sulfonate (EMS) and Tee 9 was isolated after ∼200 generations of DE of the mutated culture (described below).

### EMS mutagenesis

AWRI 796 clone 5 was treated with EMS based on Fink (1970) as follows. Cells from a YEPD culture (25 ml; 2 × 10^8^ cells ml^–1^) were collected by centrifugation (2236 rcf, 5 min) and washed twice in 15 ml of 0.1 M sodium phosphate buffer pH 7 (2.16 g NaH_2_PO_4_ and 6.54 g of Na_2_HPO_4_ made up to 200 ml with H_2_O). Cells were resuspended in fresh buffer to 15 ml (2 × 10^8^ cells ml^–1^). A volume of 1 ml was untreated (control) and used for cell enumeration and cryostorage. The remainder was treated with 0.63 ml EMS and incubated at 30°C. Samples (1 ml) were collected every 10 min, the EMS inactivated with an equal volume of freshly prepared 5% sodium thiosulphate. Following centrifugation, the cell pellet was washed once before resuspension in YEPD with 26% glycerol for cryostorage and colony enumeration (duplicate samples). Relative survival was calculated from the mean viable cell count. Cells treated with EMS for 50 min (T50; representing 60% survival) provided the starting material for DE.

### DE experiment targeting improved fructose utilization

The DE experiment used a continuous culture approach (Zeyl 2004) and was pursued in two stages. The first sought to define a fructose concentration in the feedstock and dilution rate that ensured limiting concentrations of fructose in the fermentation vessel and maintained a largely stable cell number (∼5 × 10^7^ ml^–1^). The second adopted these conditions and proceeded with the extended evolution experiment.

**Figure 1.**
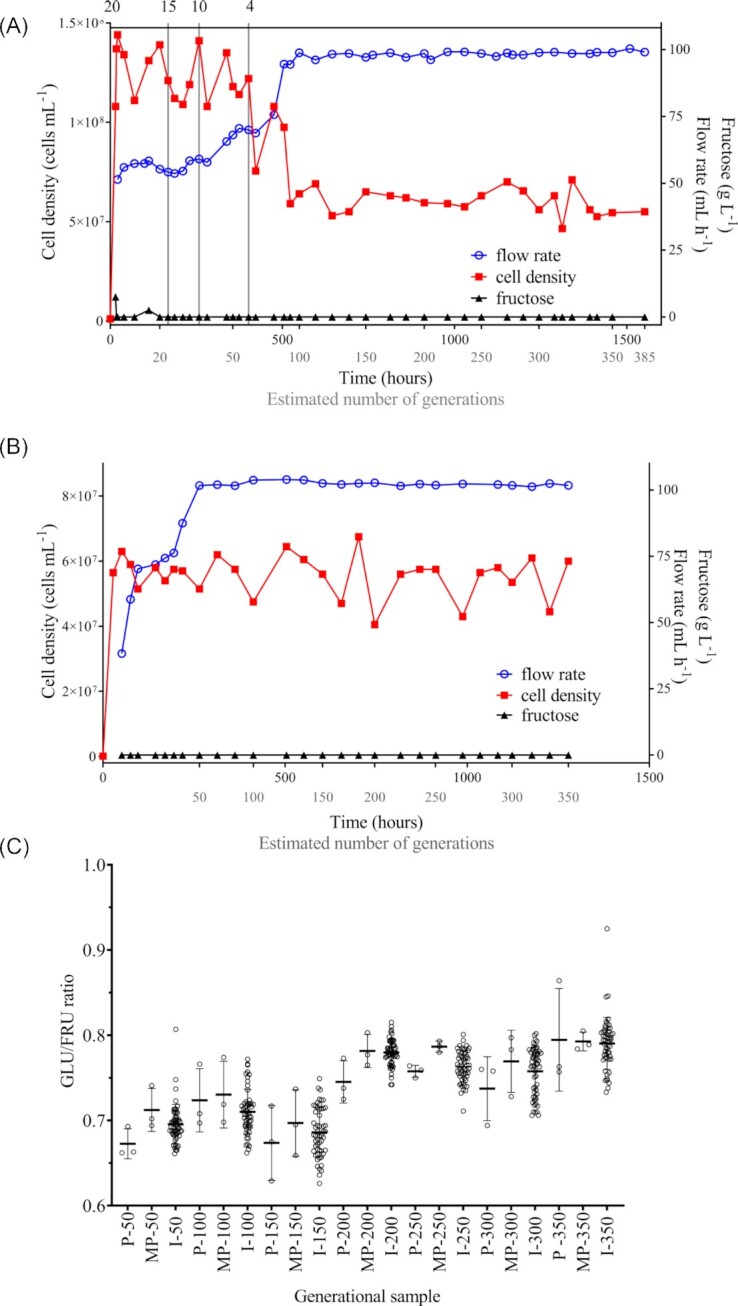
DE of AWRI 796 for improved fructose utilization in CDGJM. Continuous culture of AWRI 796 (T50) was undertaken in a CDGJM with fructose (CDGJM_F) in two separate DE experiments to firstly establish the dilution rate and fructose concentration of the feedstock to obtain a stable target cell density of ∼ 5 × 10^7^ cells ml^–1^ for adaptive evolution **(A)** and secondly, to generate isolates with increased fructose utilization **(B)**. Residual fructose, flow rate, and cell density were measured throughout the experiments. A total of 54 isolates within each approximately 50-generational sample were evaluated for sugar utilization (glucose and fructose) in CDGJM. The evolving population was monitored over the ∼350 generations according to their fructophilicity **(C)**. The GLU/FRU ratio for each individual was calculated from the Area Under the Curve (AUC) of utilization for each sugar and plotted with the mean ± SD. Each generational sample included the parent AWRI 796 (denoted by ‘P-’), the mixed population (denoted by ‘MP-’) and individual isoaltes (denoted by ‘I-’). Statistical analysis of the data was via 1-way ANOVA and Tukey's multiple comparisons tests at *P* = 0.01; see File S1, Supporting Information).

The experiment was conducted in a BIOSTAT^®^ A plus (Sartorius BBI System GmbH, Germany) equipped with a 1-l fermentation vessel and controlled using the MFCS/DA A plus 2.1 software (Sartorius BBI System GmbH). EMS treated cells (T50), were streaked from the glycerol stock onto YEPF agar and grown overnight at 30°C. A loopful of yeast (many colonies) was subsequently inoculated into liquid YEPF and grown overnight prior to inoculation at 1 × 10^5^ cells ml^–1^ in 500 ml CDGJM_F (4 g l^–1^ fructose) in the bioreactor. The medium was supplemented with ergosterol (10 mg l^–1^) and Tween 80^®^ (0.5 ml l^–1^) to supply sterols and fatty acids for cell membrane synthesis under anaerobic conditions (Ribéreau-Gayon et al. 2006). The vessel was maintained at 30°C, continuously agitated (200 rpm) and fitted with a water-filled air lock. The CDGJM_F feedstock initially contained fructose at 20 g l^–1^ and was supplied at 50 ml h^–1^ (2.13 g l^–1^ h^–1^ fructose). Inlet and outlet flow rates matched to maintain a constant culture volume of ∼500 mL. The fructose content of CDGJM_F was progressively reduced and the dilution rate increased (Fig 1A).

In the subsequent evolution experiment, fermentation was again initiated as a batch culture in CDGJM_F4 using a fresh overnight culture of EMS treated cells (T50), and after 24 h (∼5 × 10^7^ cells ml^–1^), proceeded as a continuous culture, with an initial dilution rate of 0.08 h^–1^. The dilution rate was progressively increased (0.12, 0.14, 0.15, and 0.18 h^–1^) over 220 h to a final value of 0.2 h^–1^ (i.e. 100 ml h^–1^), which was held for the duration of the experiment (Fig. 1B). Fructose concentration in the exhaust medium was measured using Clinitest^®^ tablets. The culture was sampled every 48 h for cell enumeration, suggesting ∼350 generations over the 1278 h (∼53 days) of the experiment. For mutant characterization, 1 ml samples were collected at 50-generation intervals, cells harvested (20 800 rcf, 1 min) and the medium replaced with YEPF and sterile glycerol added (to 15%) for cryostorage.

### Microscale (0.2 ml) screen for fermentation performance

Glycerol stocks were streaked onto YEPD agar for single colonies. A total of 54 colonies per time-point were inoculated into deep 96-well plates containing 0.5 ml YEPD, and incubated statically overnight at 30°C. AWRI 796 and the mixed evolved population were included as controls. For each isolate, a 10-ml tube containing 1 ml starter medium with 25 g l^–1^ of each of glucose and fructose (CDGJM_G+F50) was inoculated with 20 μl of YEPD culture and incubated (30°C, static) until stationary (∼10^8^ cells ml^–1^). Each 1 ml culture was then diluted with 4 ml CDGJM_G+F100 (50 g l^–1^ glucose and 50 g l^–1^ fructose) and 10 μl inoculated into a 96-well plate well containing 190 μl of CDGJM_G+F100 (∼10^6^ cells ml^–1^). The 54 isolates were arranged between three ‘source’ plates, each being was divided into four quadrants (each with 18 isolates plus the parent and MP) and, thus quadruplicates. On column of wells was left uninoculated (sterility control), whilst another remained empty to allow for addition of standards during later analysis of fructose and glucose. A total of six replicates of each source plate were made using a liquid handling robot (Tecan EVO 150), to allow for sacrificial sampling for monitoring of the fermentation (Liccioli et al. 2011b). Plates were sealed with Breathe Easy gas permeable membrane (Diversified Biotech, Boston, MA, USA) and incubated at 20°C, 75% humidity, < 1% O_2_ concentration (nitrogen gas). Every 24 h, a single replicate plate was removed and frozen (−20°C) for later enzymatic determination of glucose and fructose.

### Laboratory scale (250 ml) fermentations of DE isolates

Fermentation performance of the DE isolates was evaluated against the parent and reference strains according to Walker et al. (2003) with minor modifications. Starter cultures (50 ml; 50 g l^–1^ glucose and 50 g l^–1^ fructose) were used to inoculate 250 ml (triplicate) fermentations in CDGJM_G+F230 (115 g l^–1^ glucose and 115 g l^–1^ fructose). A Medicel Explorer bioreactor system (Medicel Oy, Finland) allowed for automatic sampling. Fermentation vessels were fitted with water-filled airlocks and kept at 30°C with constant agitation (magnetic stir bars, 200 rpm). The headspace was continuously flushed with filtered nitrogen (0.45 µm, 5 ml min^–1^). Samples (3 ml) were collected and chilled (−5°C) to prevent metabolic activity, with permanent storage (−20°C) prior to analysis. Sampling frequency varied from 6 to 8 h during the early and final stages, to 12 hourly mid-fermentation.

### Measurement of sugars, nitrogen, and metabolites

Clarified supernatant (20 800 rcf, 2 min) was used for metabolite analysis. Residual glucose and fructose were measured enzymatically (Boehringer-Mannheim 1989) with volumes adjusted to 100 μl for microtiter plate analysis (Walker et al., 2014). A liquid handling robot (Tecan EVO 150) collected samples for absorbance readings using an Infinite® 200 PRO microplate reader (Tecan Group Ltd). Samples were diluted 1 in 10, and 1 in 100 for analysis (Walker et al. 2014).

Other analyses included nitrogen by spectrophotometry (Dukes and Butzke 1998) using the Primary amino acid nitrogen (PAN) kit (K- PANOPA; Megazyme), and major metabolites (organic acids, glycerol and ethanol) by HPLC (Lin et al. 2020).

Data was organized, analyzed and graphed using GraphPad Prism software (versions 8.0.0 and 9.0.0 for Windows; GraphPad Software, San Diego, www.graphpad.com). Area Under the Curve (AUC) calculations and statistical analysis using One-way Analysis of Variance Analysis (ANOVA) and multiple comparisons testing was also with GraphPad Prism.

### Evaluation of [^14^C]-fructose uptake

To specifically monitor fructose transport, uptake of [^14^C]-fructose was measured according to Schneider and Wiley (1971). Triplicate cultures of Fermichamp® (fructophilic wine strain), the parent AWRI 796 and evolved strain, Tee 9, were grown in CDGJM_G+F230 with 600 mg l^–1^ N. Cells were collected from the fermenting cultures at 16.91 (AWRI 796), 0.72 (Tee 9) and 0.02 g l^–1^ (Fermichamp®) total sugar, respectively. Dry Cell Weight (DCW) represented the weight of 10 ml of culture on a preweighed 0.22-µm cellulose acetate filter disk (Ø 47 mm, Whatman) when microwave dry (300 W; 5 min). For the experiment, 1.3 ml of culture was washed and resuspended in 0.02 M KH_2_PO_4,_ to which 2.5 μl of radiolabelled [^14^C] D-fructose (Bioscientific – 50 μCi, specific activity 9.25–13.3 GBq/nmol) were added. Cultures were constantly agitated and samples (20, 60, 150, and 300 s intervals) washed twice with 0.02 M KH_2_PO_4_ on 0.22-µm filters, and placed into scintillation vials containing 4 ml of scintillation fluid (Starscint, 6013248). The amount of radiolabelled D-fructose retained by harvested cells was determined using a Parkard scintillation counter, counting ^14^C CPM for 2 min. Fructose uptake rate (nmol mg^–1^ DCW) was determined from a calibration curve ([^14^C] D-fructose spiked filtered fermentation supernatant) and DCW values (data not shown).

### Whole-genome characteristics and polymorphic analysis of wine yeast

#### Genome sequencing

Fermichamp® (fructophilic wine strain), AWRI 796 and the evolved strain, Tee 9 were grown overnight in 10 ml YEPD (30°C; 160 rpm). Cells were pelleted (4500 rcf, 2 min), and sent to the Australian Centre for Ecogenomics at the University of Queensland (Brisbane, Australia) for DNA extraction and genome sequencing. Libraries were prepared using Illumina's Nextera XT library preparation kit, with NGS sequencing using MiSeq v3 technology producing an average of 5 million pair-end reads per sample (300 bp coverage).

The fastq sequence data (as GZ files) are available upon request. AWRI 796 isolate (MP2_S2_L001_R1_001.fastq.gz, MP2_S2_L001_R2_001.fastq.gz), Tee 9 (MP2_S1_L001_R1_001.fastq.gz, MP2_S1_L001_R2_001.fastq.gz), and Fermichamp*®* (MP2_S3_L001_R1_001.fastq.gz, MP2_S3_L001_R1_001.fastq.gz).

### Alignment and SNP/InDel variant calling

Reads were aligned using the Burrows–Wheeler Aligner (BWA/0.7.15) MEM algorithm (Li 2013) to the SacCer3 reference genome (SGD R64-2-1; http://sgd-archive.yeastgenome.org/sequence/S288C_reference/genome_releases/), then sorted and indexed using SAMtools/1.9 (Li, 2013). Duplicates were marked and removed using Picard tools (picard/2.6.0), resorted and indexed using SAMtools, and the InDels realigned using the GATK/3.7 package. Variant calling analysis used freebayes/ 1.0.2-6-g3ce827d (Garrison and Marth 2012) with modified arguments (–pooled-discrete –pooled-continuous –report-genotype-likelihood-max –allele-balance-priors-off –min-alternate-fraction 0.1) and LoFreq/2.1.2 (Wilm et al. 2012) in a paired mode with their genetic ancestor (aka parent). Called variants were subsequently filtered for uniqueness against their genetic ancestor(s) using bedtools/2.26.0. The variants were filtered for quality using bcftools/1.9 (Table 1) before being annotated (Pashkova et al., 2013) and manually inspected for validity using the Interactive Genomics Viewer (IGV); Robinson et al. (2011).

**Table 1.** Variant caller and filter parameters used.

Variant caller	Variant filter parameters
Freebayes	MQM>30 & MQMR>30 & QUAL>20 & INFO/DP>40 & (SAF+SAR)>4 & (SRF+SAF)/(INFO/DP)>0.01 & (SRR+SAR)/(INFO/DP)>0.01
LoFreq	QUAL>20 & DP>20 & (DP4[2]+DP4[3])>4 & (DP4[0]+DP4[2])/(DP4[0]+DP4[1]+DP4[2]+DP4[3])>0.01 & (DP4[1]+DP4[3])/(DP4[0]+DP4[1]+DP4[2]+DP4[3])>0.01
GATK's Haplotype Caller	QD < 2.0 || FS > 60.0 || MQ < 40.0 || MQRankSum < -12.5 || ReadPosRankSum < -8.0

### Copy number and rearrangement analysis of chromosomes

Two methods were used to analyze the Copy Number Variation (CNV) of the chromosomes.

#### Method 1 (original analysis)

Using 1000 base pair sliding windows (IGVtools), normalized by the mean total read depth across the genome (GATK/3.7), the copy number across the genome was plotted and manually inspected for changes in copy number with a sample's genetic ancestor. Copy number change breakpoints were manually inspected to determine the type of rearrangement using split and discordant reads, generated with BWA mem, SAMBLASTER/0.1.24 (Faust and Hall 2014), and SAMtools.

#### Method 2 (reanalysis of AWRI 796 isolate)

Raw reads for MP2_S2 (AWRI 796 isolate) were *de novo* assembled using MEGAHIT v1.2.9 with parameters “–no-mercy –prune-level 3 –min-count 5” (Li et al., 2015, 2016). The contigs were corrected and scaffolded using the S288C reference genome SGD R64-3-1 (http://sgd-archive.yeastgenome.org/sequence/S288C_reference/genome_releases/) using ragtag v 2.01 (Alonge et al., 2019). Contigs less than 25 000 bp were removed from the assembly leaving 17 scaffolds (16 nuclear chromosomes and 1 mitochondrial genome). MP2_S2 raw reads were mapped back to the *de novo* assembled genome using BWA-mem2 v2.0, SAMBLASTER v0.1.24 and SAMtools v1.7 (Li et al. 2009, Faust and Hall 2014, Md et al. 2019).

The CNVkit batch command was used on the resulting bam with a 10 000-bp target size and in whole genome sequencing (wgs) mode (Talevich et al., 2016). Raw reads for accession number SRR2967854 (AWRI 796) from the SRA (https://trace.ncbi.nlm.nih.gov/Traces/sra/?run=SRR2967854) using SRA toolkit v2.10.9. The reads were mapped and ran through CNVkit using the same parameters as before. Raw reads for the reference genome S288C were ran through the pipeline to ensure consistency. The resulting bams were compared within the genome browser, IGV (Robinson et al. 2011) on chromosome 1 to evaluate whether there was significant strain divergence between the sequence of the isolate used in this study (MP2_S2) and the published sequence SRR2967854. Finally, Sourmash was used to validate sequence divergence of the fastqs *via* sequence containment within the *de novo* assembled genome (Pierce *et al*. 2019).

### Allele frequency analysis

The previously processed alignments were used to call variants using GATK's Haplotype Caller, then filtered using the parameters listed in Table 1. The filtered variant calls were used to generate allele frequency calls across the genome using an in-house script, which were subsequently plotted. Loss of heterozygosity (LOH) events were manually called using visual inspection.

### Protein prediction software

Protein prediction SNAP2 software (https://www.rostlab.org/services/SNAP/; Hecht et al. 2015), mutfunc (http://mutfunc.com; Wagih et al.^2018^), and PROVEAN (http://provean.jcvi.org/seq_submit.php; Choi et al. 2012) were used to predict putative deleterious amino acid substitutions within mutant gene sequences. Homology modelling and sequence comparison between related proteins used Phyre2 software (Protein Homology analogY Recognition Engine version 2; http://www.sbg.bio.ic.ac.uk/Phyre2; Kelley et al. 2015).

### Classification and numeric enrichment of identified genes annotated to Gene Ontology (GO) terms using computational software tools

Two datasets representing genes with nonsynonymous SNPs (nsSNPs) were separately analyzed: the total 180 genes and the 98 predicted to be deleterious to protein function. Hierarchical clustering and over-representation (enrichment) of genes based on shared Gene Ontology (GO) terms was performed SGD GO Slim Mapper (https://www.yeastgenome.org/goSlimMapper) and using GO Finder Version 0.86 software (https://www.yeastgenome.org/goTermFinder) respectively, or together, with ShinyGO v0.741software (http://bioinformatics.sdstate.edu/go/; Ge et al. 2020).

### Allelic swaps in AWRI 796 using CRISPR/cas9 gene editing

Single nucleotide changes (C/G>T/A) were introduced *via* CRISPR/Cas9 to create the amino acid changes Gid7 (E726K) and Fba1 (G135S) as single homozygous mutations in the diploid genome. The two-plasmid system described by Shaw et al. (2019) centered on the guide RNA plasmid, pWS082 and CRISPR/Cas9 expression plasmid, pWS173 and a DNA template with the mutation. Plasmids were bought from Addgene (https://www.addgene.org/guides/crispr/) and propagated in *Escherichia coli* NEB5-alpha prior to extraction (Wizard Plus SV Minipreps DNA Purification System; Promega).

Guide RNA sequences were designed to the *FBA1* (*YKL060C*) and *GID7* (*YCL039W*) genes in the AWRI 796 genome (taxid:764097) with Benchling ([Biology software] 2020. Retrieved from https://www.benchling.com/; Lee et al. 2015). These sequences were oligomers (Table 2) that were annealed together and cloned into plasmid pWS082. The recombinant plasmids pWS082_FBA1 and pWS082_GID7 were confirmed by Sanger sequencing (AGRF, Adelaide) using primers pWS082seq (Table 2) and DNA sequence analysis using BLAST software (https://blast.ncbi.nlm.nih.gov/Blast.cgi; Altschul et al. 1990) and/or Clustal Omega (Electronic Source: Clustal Omega; Sievers et al. 2011).

**Table 2.** Primers used in this study.

Oligomer primers	Sequence 5’–3’
sgFBA1 F	gactttGAAGCTTACTTCAAGGAACA
sgFBA1 R	aaacTGTTCCTTGAAGTAAGCTTCaa
sgGID7 F	gactttAAAGAAATGTTTGCTTCTGG
sgGID7 R	aaacCCAGAAGCAAACATTTCTTTaa
HDR FBA1 forward	CATGGTTCGATGGTATGTTGGAAGCTGATGAAGCTT ACTTCAAGGAACAC**A**GTGAACCATTATT
HDR FBA1 reverse	ATCGGTTTCTTCAGACAAATCCAACATGTGGGAGGA GAATAATGGTTCAC**T**GTGTTCCTTGAAG
HDR GID7 forward	AACCCATGGGGAAAAATTGTAACGTAGTTGCATCAA ACCCTGCAGATAAA**A**AAATGTTTGCTTCTG
HDR GID7 reverse	AATTTTCCAGATTTTTATCTTACCGTCATCACCGCCAG AAGCAAACATTT**T**TTTATCTGCAGG
FBA1 (YKL060C)_A^a*^	GCATCCTCTCTTTCCATATCTAACA
FBA1 (YKL060C)_D^a*^	CGAAGAGTTCCAGAATGAAATAAAA
GID7 (YCL039W)_A^a*^	GTTTCAGATCTATGCTGAGACACG
GID7 (YCL039W)_D^a*^	CCATTTGGTATGGATTATCACTAGG
pWS082seq^b^	GTCATCTGGAGGTCCTGTGTTC
GID7 E726K seq^b^	TGGACAGAAGCAACAGCAC
FBA1 G135S Seq^b^	GCTTACTTCGCTGGTAAGG

Primers were sourced from Sigma Aldrich (Australia) as 100 μM μl^–1^ stocks (0.05 μM scale, desalted). ^a^Primers for amplification of genes for sequencing. ^b^Primers also used for sequence confirmation of CRISPR/Cas9-derived constructs. *Primer sequences were from the Saccharomyces Genome Deletion Project (http://wwwsequence.stanford.edu/group/yeast deletion project/Deletion primers PCR sizes.txt). HDR, Homology Directed Repair. sg, single stranded guide. Bold font represents nucleotide change, whilst underlined font represents overlapping sequence in construction of double stranded DNA mutation templates. Lower case font represents nucleotides to reconstruct BsmBI site in the CRISPR/Cas9 sg RNA in the plasmid pWS082 (Lee et al. 2015).

Each double-stranded DNA template (with the single nucleotide mutation, G > A), was amplified using VELOCITY DNA polymerase (Bioline) with 8 μl of each set of overlapping homology-directed repair (HDR) oligomers (100 μM μl^–1^; Table 2) and gel purified using the Wizard® SV Gel and PCR Clean-Up System (Promega). The mutation was introduced into the yeast genome by HDR. AWRI 796 yeast cells were transformed using the lithium acetate method (Gietz et al. 1992) with a reaction mixture containing the plasmids pWS082_FBA1 or pWS082_GID7, pWS173, and the mutation template. G418 resistant transformants were picked and cured of plasmid by growth on nonselective media (YEPD, 3 days at 28°C), with plasmid loss confirmed as growth sensitivity to G418. Genomic DNA extracted using phenol–chloroform/glass beads (Adams et al. 1997) and used to amplify the mutation sequences for *GID7* and *FBA1* genes with VELOCITY DNA polymerase (Bioline) using gene specific primers (A and D). The gel purified DNA was sequenced using GID7 E726Kseq and FBA1 G135Sseq (Table 2) and compared with the wild type DNA (AWRI 796) using CLUSTAL Omega software.

### Laboratory scale (100 ml) fermentations of Gid7 (E726K) and Fba1 (G135S) mutants

Triplicate fermentations were performed in 100 ml of CDGJM (with variable nitrogen, as described below) or Semillon juice using 250 ml Erlenmeyer flasks fitted with water-filled airlocks and sampling ports.

Single colonies were cultured in 25 ml YEPD (24 h, ambient temperature, 140 rpm). The cultures were inoculated into 50 ml of starter media at 2.5 × 10^6^ cells ml^–1^ and regrown. For fermentation set up, the starter culture was centrifuged and washed with identical media to that used for the fermentation trial. Fermentations (triplicate) were inoculated at 5 × 10^6^ cells ml^–1^, incubated at 18°C with shaking (200 rpm), and monitored by refractive index (°Brix). Samples (1 ml) were collected daily and the supernatants stored for later analysis (described above) and considered dry when total sugar was < 2.5 g l^–1^ as determined by AIMTAB Reducing Sugar Tablets (Rowe Scientific, Adelaide). DCW from 5 ml of culture was determined as described above.

### Fermentation of CDGJM with various total N

CDGJM_G+F230 was prepared with various N (90, 250, or 400 mg l^–1^ total N) using a 25x amino acid stock (Table S1, Supporting Information). These N values equated to 83, 230, and 368 mg l^–1^ YAN, respectively. CDGJM Starter medium contained 50 g l^–1^ glucose, 50 g l^–1^ fructose; 450 mg l^–1^ total N, 10 mg l^–1^ ergosterol, and 540 mg l^–1^ Tween 80^®^.

### Fermentation of Semillon juice

The juice used was produced from a 2016 Semillon (Coombe Vineyard, University of Adelaide), which was handpicked, processed and immediately frozen in a 5 l plastic container. The juice parameters were 233.91 g l^–1^ sugar (as glucose and fructose), 96 mg l^–1^ YAN, TA 4.9 g l^–1^, and pH 3.18). Prior to use, the juice was thawed at 2°C and filtered (0.45 and 0.22 μm nitrocellulose). Starter medium was an equal volume of YEPD and Semillon juice.

### Cytometry

Cell number and viability were measured via flow cytometry with propidium iodide (PI) using and a Guava 12HT system and Guava easyCyte^TM^ software (Millipore). Samples were serial diluted in phosphate buffered saline (PBS) with 10% of PI, which was made fresh from a stock solution (1 mg ml^–1^). Fluorescence attributed to PI (PI positive and negative) was gated on FL3. Total cells, viable cells (PI negative events) and dead cells (PI positive events), and cells per ml were tabulated using Microsoft Excel 2013 software.

## Results

### Adaptive evolution of a clonal isolate of AWRI 796

A single clone of AWRI 796 (clone 5) was isolated and evaluated as a physiological representative of the average population from the commercial package (data not shown). This isolate provided the genetic background for selective improvement of fructose utilization, a reference for the characterization of the evolving population, and was the ancestral strain (parent) in genomic analysis. As genetic variation is key to adaptive evolution (Chambers et al. 2009), random mutations were introduced into the genome of clone 5 using EMS, to increase genetic heterogeneity, and the probability of isolating beneficial mutants. The T50 culture (EMS, 50 min, 60% survival) was used for the study.

A preliminary bioreactor experiment was undertaken to establish the conditions for continuous culture to establish a cell density to allow for adaptive evolution (Fig. 1A). This was followed by a second bioreactor experiment to generate isolates with increased fructose utilization (Fig. 1B).

In the first experiment, the bioreactor contained 500 ml of CDGJM_F4 (4 g l^–1^ fructose) and ∼1.5 × 10^8^ cells ml^–1^ before fructose was supplied at 2.13 g l^–1^ h^–1^ and a dilution rate of 0.1 h^–1^ (i.e. 20 g l^–1^ feedstock, 50 ml h^–1^ flow rate). Over the first 420 h, the fructose concentration was incrementally decreased in the feedstock (20, 15, 10, and 4 g l^–1^) whilst the dilution rate increased to 0.2 h^–1^ until a target of ∼5 × 10^7^ cells/ml was reached. This setting allowed the cell density to be maintained until the end of the experiment (1552 h). Whenever sampled, the exhaust medium contain no detectable fructose, except initially when the 20 g l^–1^ feedstock was used (Fig. 1A). Nitrogen (600 mg N l^–1^ as amino acids and ammonium chloride) and ‘anaerobic factors’ ensured that only fructose was limiting, allowing the cells to rapidly consume the available sugar.

The actual DE experiment was undertaken using the T50 culture grown from glycerol stock as before, rather than a transfer of culture from the first experiment. The 500 ml batch culture attained 5 × 10^7^ cells ml^–1^ after 24 h, when the continuous culture phase was started with supply of CDGJM_F4 (0.08 h^–1^ dilution rate, 40 ml h^–1^ flow rate; Fig. 1B). Over the next 220 h the dilution rate was progressively increased to 0.2 h^–1^ (100 ml h^–1^ flow rate), where it was held for the duration of the experiment (1278 h). During this time, the cell density fluctuated between 4.05 and 6.75 × 10^7^ cells ml^–1^. Fructose was undetectable in the fermentation vessel (Fig. 1B), indicating rapid consumption of supplied fructose.

Given an estimate (Zeyl 2004) of the rate of adaptive mutation (1 per 10^11^ cell divisions; ∼40 generations) and allowing for additional divisions to achieve significance in a population, the culture was sampled seven times at ∼50 generation intervals (Fig. 1B), to allow for emergence and identification of a beneficial mutant. Previously, we reported that beneficial phenotypes were observed after ∼250 generations (∼270 days) under a sequential batch culture scenario (McBryde et al. 2006). By comparison, continuous culture (with an exponential culture) used here, reduced the evolutionary experiment timeframe to only 53 days to achieve 350 generations at a dilution rate of 0.2 h^–1^ and average population of ∼5 × 10^7^ cells ml^–1^.

### Screening of the evolved population in microscale fermentations

The evolving population was sampled every 50 generations and evaluated as single colony isolates for fermentation improvement using a high-throughput screening approach (Liccioli et al. 2011b). A total of 378 isolates (54 per population; 7 populations) were evaluated in 21 microfermentation (0.2 ml, 96-well plate) screens, with each fermentation performed in quadruplicate with 18 isolates per plate. AWRI 796 and the corresponding ‘mixed population’ (MP) were included in each plate as controls. The isolates were evaluated in CDGJMG_G+F100 (50 g l^–1^ each of glucose and fructose) with 600 mg N l^–1^ (551 mg YAN l^–1^) rather than that of typical fermentation screens (≥ 200 g l^–1^ sugar, ≤ 450 mg N l^–1^), which mimic wine fermentation (Walker et al. 2014, Peter et al. 2018b). Whilst N was kept constant, the sugar had been increased from the DE culture to determine whether the evolved strains had lost their ‘fitness’ for high-sugar batch fermentation.

Fermentation performance varied between the isolates. Figure S1 (Supporting Information) shows the results from a typical batch, with a few isolates fermenting faster than the parent, while others were slower. Although the former suggest a possible improvement in fructose utilization and overall fermentation in some isolates, the resolution of the fermentation curves was insufficient, thus differences were assessed using Area Under the fermentation Curve (AUC) values, which bidimensionally represent residual sugar vs. time (Liccioli et al. 2011a). The ratio between the AUC values for glucose vs. fructose utilization, i.e. the GLU/FRU ratio, was then calculated as a measure of an isolate's ability to use fructose compared to glucose independent of overall fermentation duration. The closer the GLU/FRU ratio to 1, the smaller the difference between the kinetics of glucose *vs* fructose utilization and hence the more fructophilic the strain.

The GLU/FRU ratios of the isolates were plotted along with the parent and MP for each generational sampling (Fig. 1C). The marked variation between plates in the GLU/FRU ratio of the parent most likely arose due to the screen being conducted over several weeks and not as a single experiment. Also, false positives/negatives and outliers were inevitable given the fermentation scale (0.2 ml) and small inoculum volume, together with variations in microenvironmental conditions (plate position, humidity, temperature, and oxygen; Liccioli et al. 2011b). While the outliers in the parental data obscured the significance of differences in early samples, a trend towards higher GLU/FRU ratios (average value, and proportion of isolates per generation) was clear at 200 generations and beyond, and alluded to the population becoming more fructophilic as the DE progressed (Fig. 1C; File S1, Supporting Information).

Using the GLU/FRU ratio, the 50 best performing isolates from across those evaluated were readily identified (File S1, Supporting Information). It was reasoned that this number of candidates for further evaluation could be reduced by excluding those with poor overall fermentation performance (OFP), despite showing increased fructophilicity. Thus, for each isolate a measure of OFP was determined as a ratio of the AUC for its total sugar (glucose and fructose) catabolism compared to that of the parent in the corresponding plate. OFP ratios of 1 or less, would indicate isolates able to complete fermentation at least as rapidly if not more so than the parent. Using these two criteria, 19 isolates were selected from the 50 shortlisted, with representatives from the following generations: 50^th^ (1 clone), 200^th^ (3 clones), 250^th^ (2 clones), 300^th^ (2 clones), and 350^th^ (11 clones). The GLU/FRU ratios of these isolates (0.79–0.84 [Ave. = 0.80 ± 0.01]) compared to the parent (0.73 ± 0.05), together with low OFP ratios (0.85–0.95 of the parent value) were indicative of overall improvement (File S1, Supporting Information).

**Figure 2.**
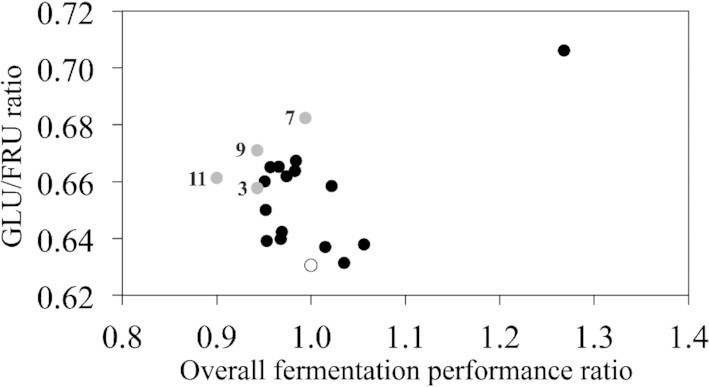
Fermentation performance of nineteen evolved yeast isolates compared to parent strain AWRI 796. Isolates were evaluated against AWRI 796 in microscale (0.2 ml) fermentations in CDGJM_G+F100 (50 g l^–1^ glucose, 50 g l^–1^ fructose, and 600 mg l^–1^ YAN). OFP relative to the parent (AUC of total sugar utilization as a ratio to the parent value) and the GLU/FRU ratios were plotted. The parent is denoted by an open circle, whilst the 19 isolates with solid circles. A total of four isolates (3, 7, 9, and 11) were subject of further study.

When assessed in a second microscale screen aimed at eliminating false positives and further narrowing the number of promising candidates for evaluation, a range in performance relative to the parent was evident (Fig. 2). Of 19 isolates previously shown to have higher GLU/FRU ratios and improved OFP, five (6, 10, 15, 16, and 19) performed worse in the second screen, having OFP ratios greater than 1. The GLU/FRU ratios of the parent was 0.63, while most isolates ranged between 0.63 and 0.71, and isolate 10 was 0.94 (Table S2, Supporting Information). These differences between the screens reiterated that while convenient, the microfermentation screen has a degree of variability. Notwithstanding this, three out of 19 isolates (3, 9, and 11) were selected as having both superior OFP compared to the parent (i.e. OFP ratios of 90%–95%) and a GLU/FRU ratio greater than 0.63. Isolate 7 was also retained because of this higher fructophilicity (0.68 vs. 0.63), despite having a similar OFP to the parent (0.99 vs. 1.0; Table S2, Supporting Information).

### Fermentation performance of evolved isolates in CDGJM

**Figure 3.**
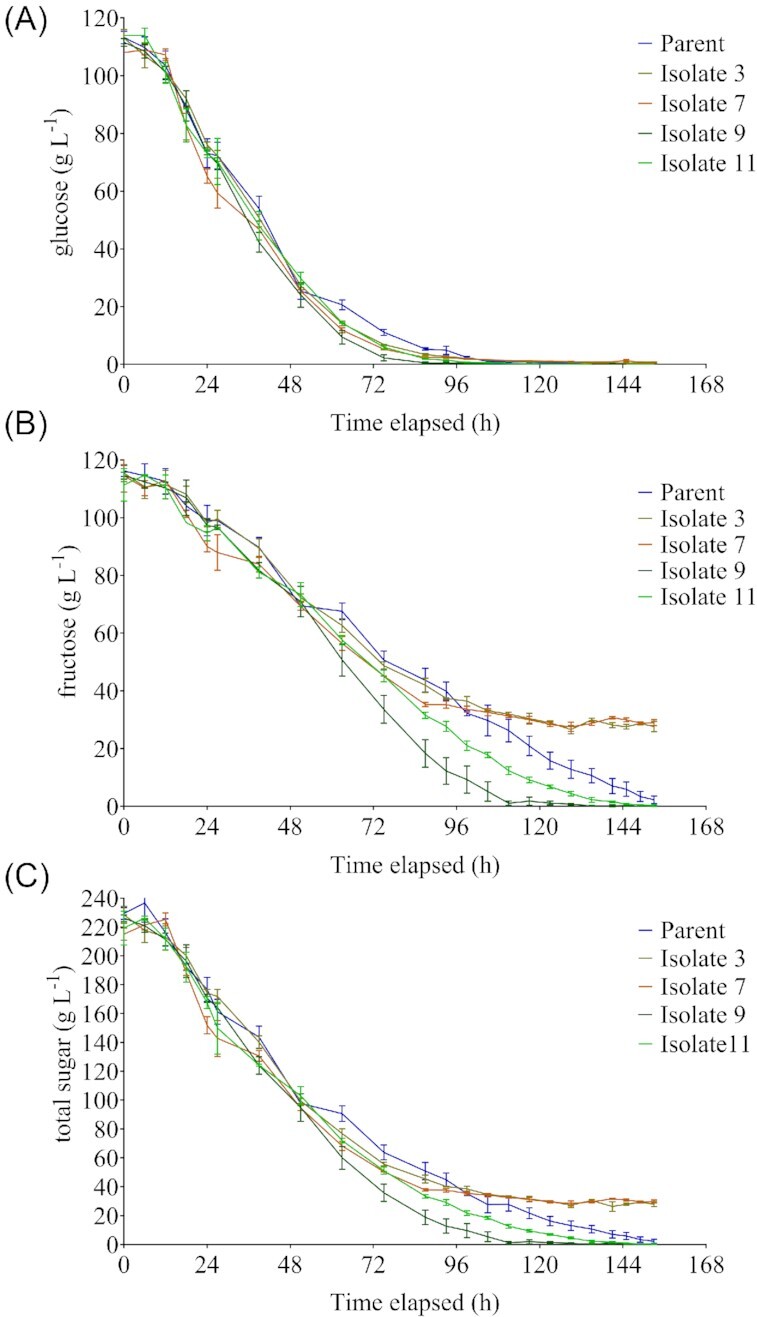
Fermentation performance of four evolved yeast isolates compared to parent strain AWRI 796. Sugar consumption curves are shown for four isolates and the parent, AWRI 796. **(A)** Residual glucose; **(B)** residual fructose, and **(C)** total residual sugars. Fermentations were conducted in 250 ml of CDGJM_G+F230 (115 g l^–1^ glucose, 115 g l^–1^ fructose). Values represent the average of three replicates (± SD).

Selected isolates were evaluated in two experiments in CDGJM_G+F230 at a larger scale (250 ml), which allowed greater precision in inoculation rates and control of environmental conditions. Isolates 3, 7, 9, and 11 were initially compared against the parent (Fig. 3), where a clear differentiation in the second half of the fermentation was observed. Isolate 9 had the quickest fermentation, depleting all sugars within 117 h compared to parent AWRI 796 (153 h), whilst isolate 11 took 135 h. Isolates 3 and 7 failed to complete, leaving ∼30 g l^–1^ of residual sugar (mainly as fructose). Glucose consumption was essentially the same between the parent and all four isolates (Fig. 3A) with differences noted in fructose consumption (Fig. 3B). The selective loss of fructose utilizing ability in isolates 3 and 7 was unexpected, but may be linked to the higher ethanol yield (11.75% by volume) of these high sugar (230 g l^–1^ total) fermentations compared to the adaptive experiment (trace ethanol). The data implies not only the adaptive evolution of isolates to improved fructose utilization (e.g. isolates 9 and 11), but also the potential, inadvertent decrease in ethanol tolerance in some isolates (e.g. 3 and 7) when ethanol is not one of the selective pressures.

Isolates 9 and 11 were next compared with AWRI 796 and two commercial strains, EC1118 and Fermichamp® under the same conditions (Figure S2, Supporting Information), revealing different extents of fermentation. AWRI 796 (3.7 ± 1.5 g l^–1^ residual sugar) and isolate 11 (3.8 ± 0.9 g l^–1^) performed similarly, whilst isolate 9 was the best performer, having only 1.2 ± 0.3 g l^–1^ residual sugar in the terminal sample, below what is considered dry (2.5 g l^–1^). Interestingly, EC1118 and Fermichamp® failed to complete fermentation, with 16.6 ± 0.8 g l^–1^ and 9.7 ± 1.4 g l^–1^ sugar, respectively. Based on this data, isolate 9 was chosen for detailed characterization.

### Increased uptake of ^14^C fructose in cells of DE isolate 9 and Fermichamp®

**Figure 4.**
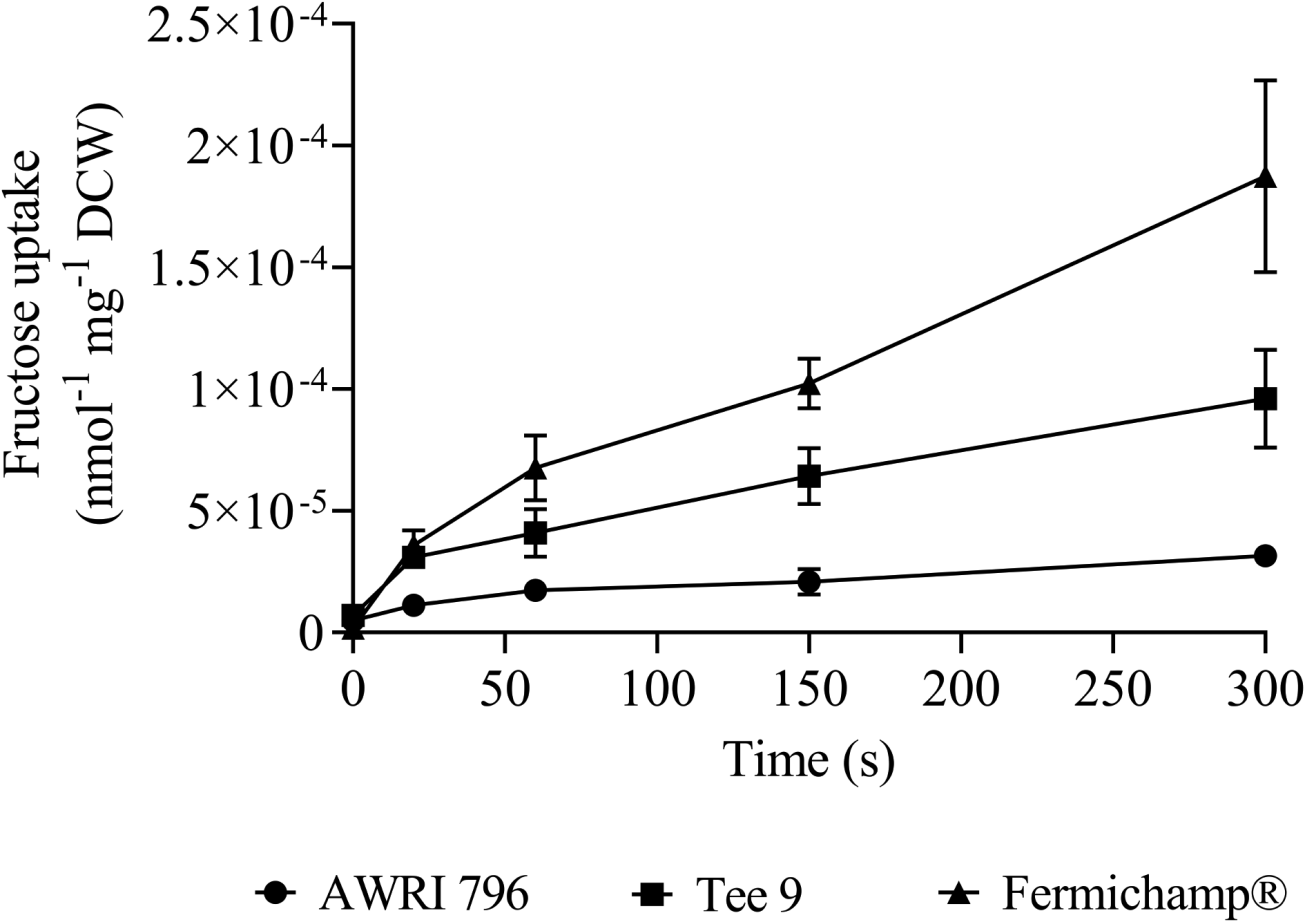
Uptake of ^14^C fructose by yeast cells from fermenting cultures in CDGJM. Triplicate cultures of AWRI796, Tee 9 and Fermichamp® were grown in CDGJM_G+F230 (115 g l^–1^ glucose, 115 g l^–1^ fructose, and 600 mg l^–1^ YAN). DCW of 10 ml of culture was determined as was uptake of [^14^C] D-fructose (50 μCi, specific activity 9.25–13.3 GBq/nmol) by the culture (1.3 ml of fermenting culture resuspended in 0.02 M KH_2_PO_4_). Washed samples (on filters) placed in 4 ml scintillation fluid were counted as ^14^C CPM for 2 min. Fructose uptake rate (nmol mg^–1^ DCW) was determined from a calibration curve ([^14^C] D-fructose spiked filtered fermentation supernatant) and DCW values (data not shown).

Isolate 9 (Tee 9) was compared to the parent strain, AWRI 796, and Fermichamp® (Oenobrand), a fructophilic *S. cerevisiae* wine strain typically used to restart high alcohol fermentations that have arrested and contain mainly residual fructose. Uptake of radiolabelled ^14^C fructose was measured using washed cells from fermenting cultures grown in CDGJM_G+F230. Samples were collected over 300 s, after addition of radiolabelled ^14^C fructose, and the amount of incorporated radioactivity, representing fructose uptake, was plotted over time (Fig. 4). A total of ∼3 × 10^–5^ nmol fructose mg^–1^ DCW were taken up after 300 s by AWRI 796, whilst for Tee 9 and Fermichamp®, the amount was ∼2- and ∼6-fold higher, respectively. These findings allude to increased fructose transport as a possible mechanism for the improved GLU/FRU ratio and fermentation phenotype of Tee 9.

### Whole genome sequence analysis to identify mutations introduced via mutagenesis and DE

The genomes of DE isolate Tee 9 and the parental strain, AWRI 796, together with Fermichamp® were sequenced using Illumina MiSeq v3 technology. Sequence comparison of our isolate of Fermichamp® to the SacCer3 reference genome (lab strain S288c) confirmed the presence of a variant *HXT3* allele reported to be responsible for enhanced fructose fermentation (Guillaume et al. 2007). Zuchowska et al. (2015) found that strains with the S288c allele took up glucose faster than fructose, whereas strains with the Fermichamp® allele utilized fructose to a greater extent. AWRI 796 and Tee 9 lacked all 10 nonsynonymous mutations seen in the Fermichamp® Hxt3p (data not shown; Zuchowska et al., 2015). The *FSY1* gene encoding the high affinity fructose H^+^ symporter (data not shown; Galeote et al. 2010, Borneman et al. 2016) was also evident in the Fermichamp® sequence but not AWRI 796. Furthermore, no similarities were found between Fermichamp® and Tee 9 that were not in AWRI 796 that could explain the improved fermentation phenotype of Tee 9 (data not shown).

For the purpose of this paper, we report only on the bioinformatics analysis of the genomes of Tee 9 and the ancestral strain, AWRI 796, which was previously sequenced to 20-fold coverage with a combination of shotgun and paired-end methods using Roche 454 (GS FLX Titanium) chemistry (Borneman et al. 2011). The authors reported AWRI 796 as largely diploid with amplification of chromosome I and a 200 kb segment of chromosome XIV detected through CNV analysis. Furthermore, AWRI 796 was mostly homozygous (with 8996 homozygous SNPs and 1041 heterozygous SNPs). The clonal isolate used in this experiment differed in that copy number variants were not found (File S2, Supporting Information), although the high similarity scores using Sourmash (Pierce et al. 2019); MP2_S2 (99.7%) vs. SRR2967854 (99.2%) in comparison to S288c (86.8%) suggested that the isolates were the same genotype.

Tee 9 was confirmed by allele frequency data as mostly homozygous and diploid, with no evidence of extensive LOH. However, 11 homozygous *de novo *SNPs were present in the Tee 9 genome, with one region on chromosome XIII containing six of these SNPSs (highlighted in File S3, Supporting Information), that may be the result of small mitotic recombination or gene conversion events (all homozygous SNPs noted in File S5, Supporting Information). These results are interesting given that low heterozygosity and extensive LOH in natural wine isolates (Peter et al. 2018a) are reportedly associated with phenotypic variation (Sampaio et al. 2019) and condition-specific fitness benefits (Lancaster et al. 2019).

In this study, there were no observable differences in chromosomal copy number (File S4, Supporting Information), alluding to a lack of large chromosomal alterations in the evolved strain, Tee 9. Whilst evolutionary processes may result in rearrangements such as insertions or deletions, translocations (Pérez-Ortín et al. 2002, Large et al. 2020), or whole chromosomal duplications as in aneuploidy (Rancati et al. 2008), the genetic variation in Tee 9 was confined to SNPs. Variant calling analysis of Tee 9 vs. AWRI 796 identified 371 mutations in the Tee 9 genome, the majority of which were G/C to A/T transitions, characteristic of the ‘mutational footprint’ of EMS (Sega 1984; File S5, Supporting Information). These mutations represented 95 noncoding mutations, and 276 coding mutations (275 codon changes and 1 indel), nearly all were heterozygous. In total, 297 genes were mutated, with 180 encoding proteins with one or more amino acid substitution (represented as 193 nonsynonymous SNPs). A total of 82 genes had synonymous SNPs (83 mutations), which did not affect the protein sequence (File S5, Supporting Information). Interestingly, none of the hexose transporters (*HXT1-17* and *GAL2*) nor glucose sensors (*SNF3* and *RGT2*; Kruckeberg 1996) were affected.

One gene (*LUC7*) had a heterozygous single nucleotide insertion (CTTTTTTG>CTTTTTTTG) resulting in a frame-shift (File S5, Supporting Information). The encoded protein, Luc7, is a component of the U1 snRNP complex responsible for the correct 5’ splicing of some 280 intron-containing genes (Parenteau et al. 2019). From that study of intron deletion (Δi) in strains grown at stationary phase or under starvation, it was proposed that introns acted to repress ribosomal protein genes (RPGs) in response to the TORC1–PKA pathway. The effect of such potentially ineffective splicing is unclear especially given that *ARP2*, *PBA1*, *RPL7A*, and *URA2*, which all have introns (Parenteau et al., 2019), also encode for protein variants in Tee 9 (File S5, Supporting Information).

Premature stop codons introduced into eight genes resulted in truncated proteins (Ndl1 (Q20*); Osh3 (Q89*), Pcp1 (W287*); Puf6 (W154*); Rrn6 (W324*); Smf3 (W123*); Tip41 (Q68*); and Tti1 (W528*). In terms of other amino acid substitutions, three algorithms, SNAP2 (Hecht et al. 2015), PROVEAN (Choi et al. 2012), and mutfunc (Wagih et al. 2018) were used to predict the impact on protein function (File S5, Supporting Information). Fba1 (G135S) together with Gid7 (E726K; File S5, Supporting Information) were chosen as initial case studies because of the link to glycolysis and fermentation (Boulton et al. 1998). The variants were evaluated as homozygous single gene mutations in AWRI 796. *FBA1* encodes fructose 1,6-bisphosphate aldolase (Fba1), which catalyses a reversible reaction in glycolysis and gluconeogenesis between fructose 1,6-bisphosphate (F1,6BP) and glyceraldehyde-3-phosphate, and is considered vital for growth. It also influences Pol III transcription when located in the nucleus, in a function separate to the aldolase activity (Cieśla et al. 2014). Prediction of functional impacts in Fba1 (G135S) using three different software was ambiguous, with the substitution predicted not to affect protein structure–function (SNAP2) or be deleterious (PROVEAN and mutfunc). *GID7* encodes a subunit of the Glucose Induced Degradation (GID) complex, which is involved in ubiquitin-proteasome-dependent catabolite inactivation of fructose-1,6-bisphosphatase (Fbp1), the enzyme responsible for dephosphorylation of F1,6BP to fructose-6-phosphate during gluconeogenesis. The GID complex is required for the negative regulation of Fbp1, and is a key point for switching between glycolysis and gluconeogenesis pathways (Regelmann et al. 2003). The exact role of Gid7 is unclear, other than its deletion results in shorter fermentation when nitrogen is limiting (Gardner et al. 2005). All three algorithms predicted Gid7 (E726K) as neutral.

### Fermentation performance of Fba1 (G135S) and Gid7 (E726K) variants in AWRI 796

Confirmation of the predicted impacts of Fba1 (G135S) and Gid7 (E726K) was sought through introduction of these variants into AWRI 796 by the two-plasmid CRISPR-Cas9 system (Shaw et al. 2019) followed by fermentation trials. Genomic DNA extracted from the parent AWRI 796 and 4 isolates per mutation provided the template to amplify the coding sequences of the two genes. Sequence comparison to the wild type gene using Clustal Omega analysis (https://www.ebi.ac.uk/Tools/msa/clustalo/; Sievers et al. 2011) confirmed the correct substitutions (Figure S3, Supporting Information). Isolate 1 for AWRI 796 Fba1 (G135S) and isolate 3 for AWRI 796 Gid7 (E726K) were then studied in triplicate fermentations in two experiments: first in CDGJM_G+F230 containing with 83, 230, or 368 mg l^–1^ YAN, and in Semillon juice (233.9 g l^–1^ sugar and 96 mg l^–1^ YAN). Comparisons were made between AWRI 796, Tee 9, and the two single gene mutants AWRI 796 Fba1 (G135S) and AWRI 796 Gid7 (E726K).

**Figure 5.**
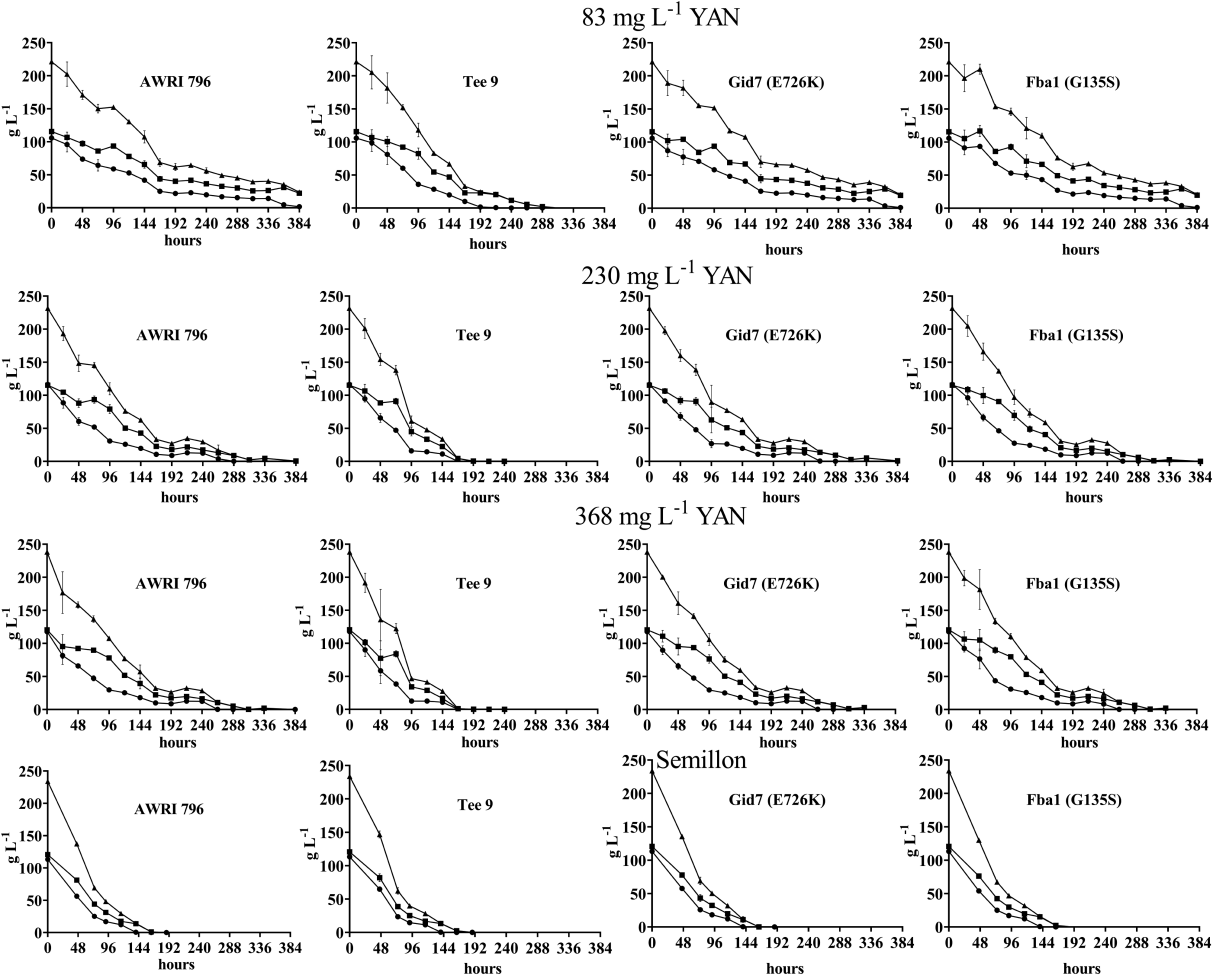
Sugar utilization curves of yeast grown in CDGJM of different nitrogen content and Semillon juice. AWRI796, Tee 9 and CRISPR mutants AWRI 796 Gid7 (E726K) and AWRI 7896 Fba1 (G135S) were evaluated through triplicate fermentations of conducted in CDGJM_G+F230 (115 g l^–1^ glucose and 115 g l^–1^ fructose) with various N contents (83, 230, and 368 mg l^–1^ YAN) or Semillon juice (113 g l^–1^ glucose, 120 g l^–1^ fructose, and 96 mg l^–1^ YAN) at 18°C. Residual glucose (•), fructose (abcde), and total sugar (▴) are shown.

**Figure 6.**
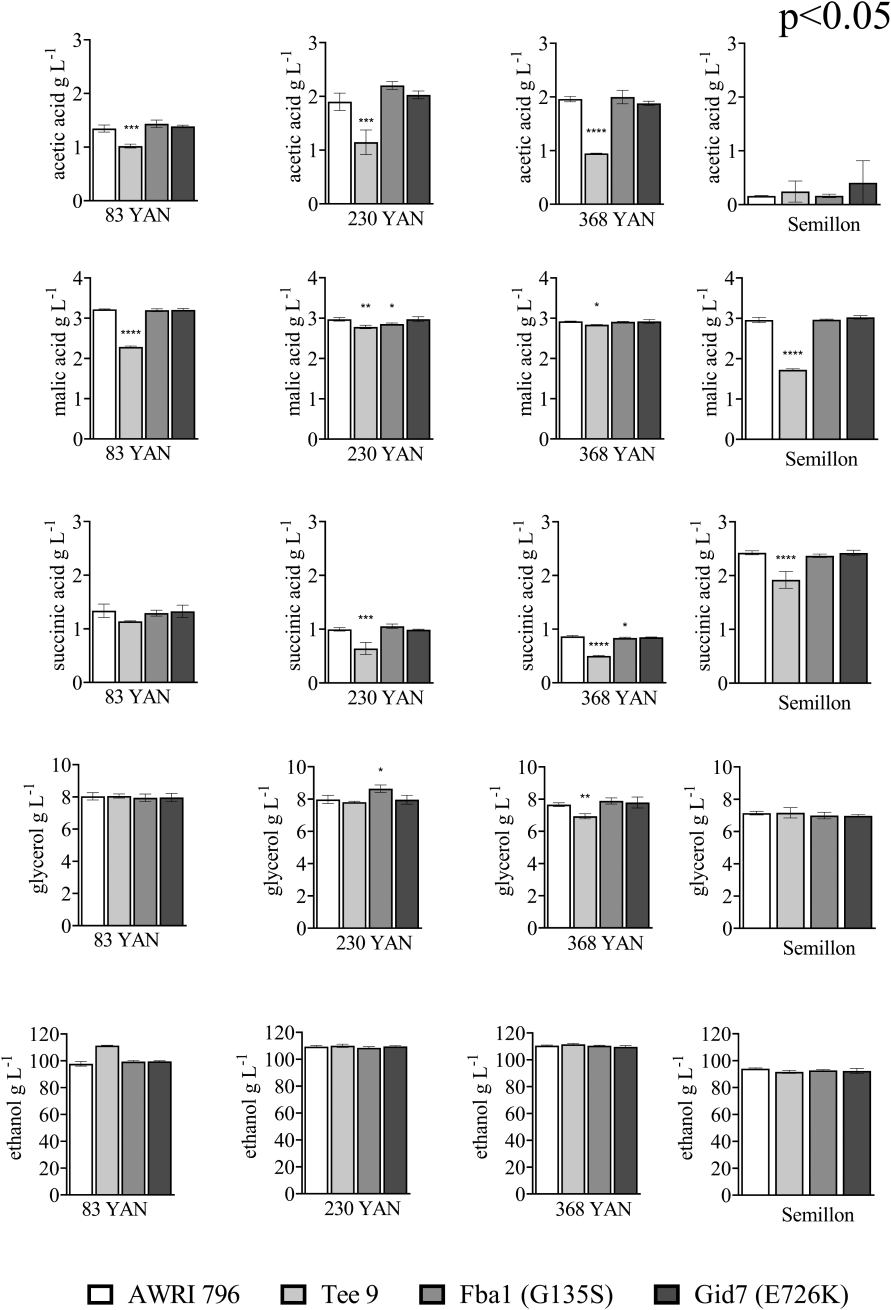
Metabolite production by yeasts grown in CDGJM with variable nitrogen and Semillon juice. AWRI796, Tee 9 and CRISPR mutants AWRI 796 Gid7 (E726K) and AWRI 796 Fba1 (G135S) were evaluated as triplicate fermentations in CDGJM_G+F230 (115 g l^–1^ glucose and 115 g l^–1^ fructose) with various nitrogen contents (83, 230, and 368 mg l^–1^ YAN) at 18°C. Fermentations in Semillon juice (113 g l^–1^ glucose, 120 g l^–1^ fructose, and 96 mg l^–1^ YAN) were undertaken separately. All strains completed fermentation under the different conditions except for CDGJM_G+F230 with 83 mg l^–1^ YAN, where only Tee 9 finished. Acetic acid, succinic acid, glycerol, and ethanol were measured by HPLC on thawed terminal samples. One-way ANOVA with Dunnett's multiple comparison testing was undertaken against AWRI 796 (*P* < .05). One asterisk (*) identifies adjusted *P-*values between .01 and .05, two asterisks (**) identify adjusted *P-*values between 0.01 and 0.001, and so on.

In CDGJM_G+F230, Tee 9 completed fermentation quicker than the parent strain, AWRI 796, regardless of YAN concentration. Mutants AWRI 796 Fba1 (G135S) and AWRI 796 Gid7 (E726K) behaved similarly to the parent, AWRI 796, under all three nitrogen conditions (Fig. 5). The ratio between glucose and fructose consumption, a measure of fructophilicity, was similar for the parent and single gene mutants, with Tee 9 having a slightly larger ratio, indicative of increased fructophilicity (Table S3, Supporting Information). Under low nitrogen (83 mg l^–1^ YAN), the parent, AWRI 796, and single gene mutants failed to complete fermentation by the time the experiment was terminated at 384 h. The residual sugar was 23.7 g ± 3.2 g l^–1^ (AWRI 796), 20.78 g ± 2.6 g l^–1^ (AWRI 796 Fba1 (G135S)), and 20.4 g ± 3.3 g l^–1^ (AWRI796 Gid7 (E726K)). Fructose represented 93.3%, 94.3%, and 94.5% of the respective residual sugar at this point. Acetic, malic, and succinic acid levels (and glycerol) were reduced in many cases with Tee 9, and whilst statistically significant are likely to only have an impact on wine sensory properties in the case of acetic acid (Fig. 6). The effect of the Fba1 (G135S) and Gid7 (E726K) mutations on these metabolites was inconsistent.

Under all conditions, Tee 9 had increased cell mortality at stationary phase compared to the other strains, which was reflected in the decreased biomass (mg/10^6^ total cells; Figure S4, Supporting Information). The work of Boer and coworkers (2008) suggests that Tee 9 may have underlying auxotrophic requirements such that when the specific nutrient runs out, the strain fails to undergo cell cycle arrest at G0/G1 and enter a resting state required for survival. Failure to regulate growth control networks such as TOR and PKA regulatory networks results in wastage of cellular glucose and ultimately in death (Boer et al. 2008).

The fermentation outcomes for AWRI 796 Fba1 (G135S) and AWRI796 Gid7 (E726K) support the predictions made from comparisons of Fba1 with other aldolase proteins using Phyre2 software (Kelley et al. 2015), where G135 could be substituted with several structurally unrelated amino acids, substantiating that it is not critical for activity. Similarly, analysis of Gid7 with other WD40 motif proteins showed E726K to be a common variant (Phyre 2; data not shown), which is consistent with the protein predictions made by the three algorithms. We conclude that whilst protein predictions may provide useful guidelines for protein structure–function relationships, they are not always reliable and open to interpretation. From the data, it is clear that the two mutations chosen, Fba1 (G135S) and Gid7 (E726K), do not affect protein function nor fermentation outcome, and that other mutations identified through SNP analysis need further investigation in a similar manner.

### Identification of potential genes associated with adaption and improved fermentation in CDGJM using GO analysis

GO analysis was undertaken to focus on other processes that could be relevant to Tee 9’s adaption and improved fermentation performance in CDGJM. The 180 genes having nonsynonymous mutations (together with the 98 predicted to be deleterious) were classified accordingly using GO Slim Mapper (SGD) and ShinyGo (Ge et al. 2020; File S5, Supporting Information). GO term enrichment analysis (GO Term Finder; SGD) highlighted genes over-represented (enriched) for GO terms related to function (*P* < 0.05). 42 out of the 180 genes were associated with ‘small molecular binding’ (SGD); 25 were predicted to have deleterious mutations, and were also associated with ‘nucleotide binding’ and ‘nucleoside phosphate binding’ (File S5, Supporting Information). Glycogen metabolism is a possible focal point for future analysis, given that four genes involved in synthesis and degradation were over-represented amongst the 42 genes.

## Discussion

Random mutagenesis in combination with DE was used to target the differential consumption of glucose and fructose during alcoholic fermentation, with the goal of increasing fructophilicity and OFP. Whilst strains such as EC1118 and Fermichamp*®* are already efficient at fructose utilization, especially under conditions leading to high ethanol content wines, the aim was not to see whether such strains could be further enhanced. Instead, the goal was to determine whether a commonly used strain with otherwise desirable winemaking properties could be improved specifically in its fermentation dynamics. AWRI 796 met the criteria, based on its fermentation performance (Liccioli et al. 2011a), and prominence in red wine production in Australia (Nordestgaard 2019). This sector alone, represents 58% of export market valued at $2.8 b (https://www.wineaustralia.com/market-insights/australian-wine-sector-at-a-glance).

Prior work demonstrated AWRI 796 to be a medium-fast fermenting strain in CDGJM containing equimolar glucose and fructose, or fructose alone (Liccioli et al. 2011a). However, it still favoured glucose consumption, having a glucose to fructose (GLU/FRU) ratio of 0.64 compared to the fructophilic wine strain, Fermichamp® (0.76; Liccioli et al. 2011a). The latter is commonly used to restart fermentations especially in high ethanol wines. The two strains differ in genetic backgrounds in terms of hexose transporters. AWRI 796 lacks the high affinity fructose transporter gene, *FSY1* (Galeote et al. 2010) present in the genomes of EC1118 and Fermichamp® (Borneman et al. 2016), as well as the variant *HXT3* allele thought responsible for the strain's superior ability to consume fructose (Guillaume et al. 2007).

The approach was a proof of concept whereby random mutagenesis was used together with DE, the originality of the experiment being the use of fructose alone as the selective condition for adaption. The genetic heterogeneity of the starting population was artificially increased by EMS treatment to enhance the likelihood of adaptive mutations sooner in continuous culture. For the DE experiment, the culture was grown in a defined medium reminiscent of grape juice in terms of phosphate, sulfate, vitamins, and minerals (Henschke and Jiranek 1993). Fructose was limiting, with ergosterol and Tween 80 as a source of sterols and fatty acids (Ribéreau-Gayon et al., 2006), and excess nitrogen (as amino acids and ammonium) added to promote cellular metabolism (Bisson, 2005). Fructose supply was regulated such that the overall concentration in the bioreactor was sufficient to maintain an appropriate cell density (Lane et al. 1999) required for DE (Paquin and Adams 1983,Wahl and Krakauer 2000, Wick et al. 2002). It was hypothesized that the most efficient fructose utilizers would to be the fittest under these conditions, thus cell density was reduced and only a low concentration of fructose supplied such that it remained undetectable in the bioreactor. These conditions were sufficient a selective pressure to generate adaptations to improve the efficiency of fructose utilization.

*Saccharomyces cerevisiae* (as an asexual population) is known to evolve as a mixture of distinct genotypes, which either coexist or undergo a temporal and dynamic succession within the population (Kao and Sherlock 2008). Given that the estimated rate of adaptive mutation is in the order of 1–10^11^ cell divisions (Zeyl 2004), the evolving population in the continuous culture was sampled at approximately 50-generation intervals. The experiment was not intended as a generational study, as neither the timeframe of the experiment, nor the evaluation of the seven generational populations was sufficiently accurate or large enough in terms of isolates to draw any conclusions. However, this sampling frequency did reveal the appearance of evolved clones with improved phenotypes from 200 generations onwards. Microscale (0.2 ml) fermentations, whilst somewhat variable because of their volume, inoculum regime, and an inability to fully control environmental influences, nevertheless allowed for 378 candidate isolates to be screened in a matter of weeks, rather than months, as with larger scale methods such as shake flasks. The number of candidates was progressively reduced through screening until only a few promising isolates needed to be evaluated in laboratory-scale (100–250 ml) fermentations.

Preliminary screening identified four isolates (3, 7, 9, and 11) with improved fructose utilization compared to the reference strain(s). The four isolates identified is a direct reflection of the number of isolates evaluated (19 rather than 50; File S1, Supporting Information) and choice of fermentation method (microscale vs. laboratory-scale); the latter being highly reproducible. It is likely that more isolates would have been identified, allowing for more than one isolate to be studied in detail. However, isolate 9 was ultimately chosen because of consistency in performance in lab scale fermentations in CDGJM_G+F230 (230 g l^–1^ sugar, variable N i.e. 83–551 mg l^–1^ YAN).

The fact that isolate 9 (Tee 9) provided no apparent advantage over AWRI 796 in Semillon juice (96 mg l^–1^ YAN 233.91 g l^–1^ sugar) suggested that the fructose utilization phenotype was condition-dependent as previously reported (Payen et al. 2016). Tee 9, was also unable to perform as well as Fermichamp®, even though it had significantly increased fructophilicity compared to its parent, AWRI 796 (Figure S5, Supporting Information).

The inability to produce a strain with greater fructophilicity than the ‘gold-standard’ Fermichamp® highlights the importance of the starting genotype. AWRI 796, lacks both the mutated *HXT3* gene identified in Fermichamp® (Guillaume et al. 2007) and *FSY1* encoding a high affinity fructose H^+^ symporter, associated with fructose utilization in *Saccharomyces spp*. yeasts such as wine strains EC1118, Fermichamp® and Uvaferm 43 (Borneman et al. 2016). Whether a such a strain would have been a better choice is a matter of conjecture, given that *FSY1* transcription is tightly regulated, being induced at low fructose or glucose concentrations, and repressed under high glucose (Galeote et al. 2010, Anjos et al. 2013) and ethanol conditions (Galeote et al. 2010). Fsy1 differs from the Hxt family (which are uniporters) as glucose is not taken up (Gonçalves et al. 2000). Interestingly, this symporter was demonstrated under experimental conditions to have variable fructose:H^+^ stoichiometry (Anjos et al. 2013). The higher stoichiometry (2 protons instead of 1 per fructose molecule) corresponded to high glycolytic flux (high sugar concentration), which in nature would be unlikely, because of transcriptional repression of *FSY1* (Anjos et al. 2013).

Variant calling analysis did not support the preliminary findings of increased fructose uptake by Tee 9 resulting from effects on hexose transport. Neither AWRI 796 nor Tee 9 possessed the 38 mutations found in the *HXT3* allelic variant (*HXT3fmp*) in Fermichamp®; including10 nonsynonymous mutations (T200A, I209V, M324I, L388M, Y389W, I392V, E414Q, G415N, I449V, and L471I) associated with enhanced fructose utilization (Guillaume et al. 2007). Those authors concluded from allelic swaps between a typical *HXT3* gene (from V5; *HXT3-V5*) and the *HXT3fmp* variant in a hexose transport deficient strain (V5 (*hxt1-7Δ*) that Hxt3p was crucial to glucose and fructose uptake in wine strains, with fructose uptake being rate limiting in the later stages of fermentation.

No mutations were identified in genes related to glycolysis (phosphofructokinase (*PFK* and *PFK2*), glucokinase (*GLK1*), hexokinase (*HXK1* and *HXK2*), nor fructose 1,6 bisphosphatase (*FBP1*)), PGI (*PGI1*) and pyruvate decarboxylase (*PDC1, PDC5*and*PDC6*), enolase (*ENO1* and *ENO2*), and phosphoglycerate mutase (*GPM1*; File S5, Supporting Information). Together with fructose 1,6-bisphosphate aldolase (*FBA1*), these glycolytic enzymes are differentially induced during the transition from respiration to fermentative (high sugar) conditions (van den Brink et al. 2008). As Fba1(G135S) was the only mutation identified amongst these genes, it was evaluated as an allelic swap in the parent strain, AWRI 796, introduced *via* CRISPR/Cas9. *GID7*, encoding the seventh subunit of the GID-deficient complex (Regelmann et al. 2003) was chosen as a second test case, given the GID ubiquitin ligase is involved in proteasome-mediated protein catabolism (including fructose-1,6-biphosphatase) and downregulation of gluconeogenesis during transition to glycolytic growth (Braun et al. 2011). Neither Fba1(G135S) nor Gid7 (E726K) as homozygous mutations could replicate the phenotypic characteristics of Tee 9. Further strain constructions are warranted to confirm whether they are indeed necessary, with the construction of a double mutant or reciprocal construction, i.e. the introduction of the AWRI 796 alleles into Tee 9, providing better insight into this.

High coverage Illumina genome sequencing together with variant calling analysis was undertaken to compare the genomes of Tee 9, its parent, AWRI 796, and Fermichamp®. We were interested to see the mutational landscape (i.e. transitions, transversions, indels, and so on; Cano and Payne 2020) in Tee 9 arising from mutagenesis and adaption to low fructose-containing CDGJM. Moreover, how the few differences between the two strains (Tee 9 *vs* AWRI796) could result in very different fermentation phenotypes, given the genetic diversity available in wine strains (Borneman et al. 2016) was also of interest. In total, 371 mostly heterozygous mutations (given the usage of a mutagen) were identified by variant calling analysis (File S5, Supporting Information). Not all are relevant to the improved phenotype since most mutations do not have a phenotype when heterozygous. Furthermore, whilst some of the mutations were beneficial, other mutations present might also cause undesirable traits unrelated to fermentation performance. One such example is Aus1(K788A) and (H935A). Aus1 is the major transporter for cholesterol and plant sterols with uptake of yeast ergosterol being low in a mutant (Papay et al. 2020). Expression is triggered under anaerobic growth when ergosterol synthesis is arrested and cells utilize exogenous sterol. The K788A mutation located within the nucleotide binding domain (782–789) abolishes ATPase activity and thus reduces cholesterol, plant sterol, and ergosterol uptake by 86%, 91%, and 50%, respectively, leading to cell death under anaerobic conditions (Papay et al. 2020). H935A, a ‘H-loop’ mutation, effects ATPase activity but not viability. The T1267I mutation identified in this study is also considered a ‘H-loop’ mutation and may have a similar effect on ATPase activity and substrate specificity. These mutations may have contributed to the lower viability of Tee 9 (Figure S4, Supporting Information).

GO analysis of genes having nonsynonymous SNPs giving rise to protein variants (File S5, Supporting Information) not surprisingly highlighted a diversity of processes and multiple gene/pathway interactions (Costanzo et al. 2010) RNAseq (Rossignol et al. 2003, Marks et al. 2008) could corroborate *in silico* predictions made by homology modelling and GO analysis, such as genes related to glycogen synthesis (*UPG1* and *GLG1*) and mobilization (*GPH1* and *GDB1*). Furthermore, *YDR277C* encoding Mth1, a negative regulator of the glucose-sensing signal transduction pathway (Lafuente et al. 2000) and *YGR014W* encoding Msb2, an osmosensor in the Sho1p-mediated HOG pathway (Hohmann, 2009), have yet to be tested in relation to fermentation. *FAT3* (required for fatty acid uptake) and *TPK1* (a cAMP-dependent protein kinase involved in Ras-cAMP signalling) are also likely candidates, as deletion of their respective paralogs, *INA1* and *TPK2*, is associated with shortened fermentation duration (Peter et al. 2018b).

The difficulty in predicting the outcome of the DE strategy, in this instance improved sugar utilization (which inadvertently is specific to CDGJM), highlights the complexity behind genome plasticity during adaption to different environmental niches. However, given the relatively small number of mutations compared to what is represented in the overall diversity of wine, industrial and wild *Saccharomyces* (Borneman, et al. 2016, Peter et al. 2018a, Duan et al. 2018), Tee 9 is still useful in genome-wide association studies whilst the collection of isolates evolved from AWRI 796 could be used in screening for other phenotypes. Ideally, the identification of one or two SNPs as causative mutations, which could be evaluated in other genotypes and used in QTL breeding programs, would allow for the transfer of a beneficial trait to produce tailored wine strains (Peltier et al. 2019). Evaluation of such variants would also better our understanding of the metabolic processes behind adaptation to fermentation stress.

## Data availability

Materials and data are within the article and supplementary data.

## Authors' contributions

M.E.W. wrote the the manuscript. T.L.W. and V.J. designed the DE experiments, T.L.W. performed the DE and evaluation of evolved isolates and genome sequencing. C.R.L.L. and M.E.W. undertook the genome analysis. S.F. reanalyzed the CNV data. M.E.W., T.A.L., and Y.B. designed and constructed yeast *FBA1* and *GID7* mutants via CRISPR, and did the fermentations. M.E.W., V.J., C.R.L.L., M.J.D., and S.F. contributed to the paper revision. V.J. is the corresponding author. All authors read and approved the manuscript.

## Authorship declaration

All authors agree that this manuscript is original, has not been published before, and is not currently being considered for publication elsewhere. We confirm that the manuscript has been read and approved by all named authors, and all have contributed significantly to the paper. The order of authors listed in the manuscript has been approved by all of us. We understand that the corresponding author is the sole contact for the editorial process and is responsible for communicating with the co-authors on the progress of the submission.

## Supplementary Material

foac022_Supplemental_Files
