# Supplementary material for: Directed evolution as an approach to increase fructose utilization in synthetic grape juice by wine yeast AWRI 796
Source: FEMS Yeast Res. 2022 Apr 26;22(1):foac022. doi: 10.1093/femsyr/foac022 (PMC9329090; doi:10.1093/femsyr/foac022)
Supplement: foac022_Supplemental_Files [file foac022_supplemental_files.zip › FEMSYR-21-07-0080.R2_SuppData.docx]

Supplementary data

**Directed evolution as an approach to increase fructose utilisation in synthetic grape juice by wine yeast AWRI 796**

**Michelle E. Walker^a#^, Tommaso L. Watson^a#^, Christopher R. L. Large^b,c^, Yan Berkovich^a^, Tom A. Lang^a^, Maitreya J. Dunham^b^, Sean Formby^d^ and Vladimir Jiranek^a,e *^**

^a^ Department of Wine Science, The University of Adelaide, Glen Osmond, SA, Australia

^b^ Department of Genome Sciences, University of Washington, 3720 15th Ave NE, Seattle,

13 Washington 98195, USA

^c^ Molecular and Cellular Biology Program, University of Washington, Seattle, Washington 98195, USA

^d^ Bioinformatics Graduate Program, University of British Columbia, Vancouver, Canada

^e^ Australian Research Council Training Centre for Innovative Wine Production, Adelaide, Australia

**Supplementary Table S1. Nitrogen composition of 25x amino acid stock**

| **Nitrogen** | **In CDGJM**  **mg L^-1^** | **% Nitrogen** | **25x stock**  **mg N L^-1^** |
| --- | --- | --- | --- |
| Alanine | 100 | 15.7 | 392.5 |
| Arginine monohydrochloride | 750 | 26.6 | 4983.8 |
| Asparagine | 150 | 21.2 | 795 |
| Aspartic acid | 350 | 10.5 | 918.8 |
| Glutamic acid | 500 | 9.5 | 1187.5 |
| Glutamine | 200 | 19.2 | 960 |
| Glycine | 50 | 18.6 | 232.5 |
| Histidine monohydrochloride monohydrate | 150 | 20.1 | 753.4 |
| Isoleucine | 200 | 10.7 | 535 |
| Leucine | 300 | 10.7 | 802.5 |
| Lysine monohydrochloride | 250 | 15.3 | 958.1 |
| Methionine | 150 | 9.4 | 352.5 |
| Phenylalanine | 150 | 8.5 | 318.8 |
| Proline | 500 | 12.2 | 1525 |
| Serine | 400 | 13.3 | 1330 |
| Threonine | 350 | 11.8 | 1032.5 |
| Tryptophan | 100 | 13.7 | 342.5 |
| Tyrosine | 20 | 7.7 | 38.5 |
| Valine | 200 | 12 | 600 |
| Ammonium chloride | 100 | 26.2 | 655 |

1 mL of 25x amino acid stock is equivalent to 18.71 mg total N or 17.19 mg YAN

**Supplementary Table S2.** Re-evaluation of the best performing 19 isolates in the second micro-scale (0.2 mL) fermentation screen.

| **Isolate** | **Generations** | **GLU/FRU ratio** | **Fermentation ratio** |
| --- | --- | --- | --- |
| Parent |  | 0.63 | 1.00 |
| 16 | 50 | 0.71 | 1.27 |
| 5 | 200 | 0.65 | 0.95 |
| 9 | 200 | 0.67 | 0.94 |
| 13 | 200 | 0.66 | 0.95 |
| 11 | 250 | 0.66 | 0.90 |
| 17 | 250 | 0.64 | 0.95 |
| 1 | 300 | 0.64 | 0.97 |
| 6 | 300 | 0.66 | 1.02 |
| 2 | 350 | 0.67 | 0.96 |
| 3 | 350 | 0.66 | 0.94 |
| 4 | 350 | 0.66 | 0.97 |
| 7 | 350 | 0.68 | 0.99 |
| 8 | 350 | 0.66 | 0.98 |
| 10 | 350 | 0.94 | 1.06 |
| 12 | 350 | 0.67 | 0.97 |
| 14 | 350 | 0.67 | 0.98 |
| 15 | 350 | 0.63 | 1.04 |
| 18 | 350 | 0.64 | 0.97 |
| 19 | 350 | 0.64 | 1.02 |

Table refers to Fig. 2.

**Supplementary Table S3. Sugar utilisation curves for yeast fermenting CDGJM with various N contents.**

Area under the curve values (± standard error) were calculated for each strain and N condition (glucose, fructose and total sugar).

|  |  |  |  |  |  |  |  |
| --- | --- | --- | --- | --- | --- | --- | --- |
|  |  |  | **83 mg L^-1^ YAN** | | | |  |
|  | Baseline (x axis) = 0 | | **AWRI 796** | **Tee9** | **Gid7(E726K)** | **Fba1(G135S)** |  |
|  | **GLU** | **Total Area** | 14253 | 9336 | 14101 | 14535 |  |
|  |  | **Std. Error** | 255.7 | 351.7 | 260.4 | 217.9 |  |
|  |  | **95% Confidence Interval** | 13752 to 14754 | 8647 to 10025 | 13591 to 14612 | 14108 to 14962 |  |
|  |  |  |  |  |  |  |  |
|  | **FRU** | **Total Area** | 21660 | 15012 | 21377 | 21830 |  |
|  |  | **Std. Error** | 228.2 | 289.5 | 230.1 | 337.5 |  |
|  |  | **95% Confidence Interval** | 21213 to 22107 | 14445 to 15579 | 20926 to 21828 | 21169 to 22492 |  |
|  |  |  |  |  |  |  |  |
|  | **TOTAL** | **Total Area** | 35913 | 24347 | 35479 | 36365 |  |
|  |  | **Std. Error** | 423.3 | 614.5 | 431.2 | 496.5 |  |
|  |  | **95% Confidence Interval** | 35083 to 36742 | 23143 to 25551 | 34633 to 36324 | 35392 to 37338 |  |
|  |  |  |  |  |  |  |  |
|  |  | **GLU/FRU** | 0.66 | 0.62 | 0.66 | 0.67 |  |
|  |  | **GLU area (% of WT)** | 1 | 0.66 | 0.99 | 1.02 |  |
|  |  | **FRU area (% of WT)** | 1 | 0.69 | 0.99 | 1.01 |  |
|  |  |  |  |  |  |  |  |
|  |  |  | **230 mg L^-1^ YAN** | | | |  |
|  |  |  | **AWRI 796** | **Tee9** | **Gid7(E726K)** | **Fba1(G135S)** |  |
|  | **GLU** | **Total Area** | 9238 | 7372 | 9230 | 9162 |  |
|  |  | **Std. Error** | 211.6 | 153 | 159.8 | 219.1 |  |
|  |  | **95% Confidence Interval** | 8823 to 9652 | 7073 to 7672 | 8916 to 9543 | 8733 to 9591 |  |
|  |  |  |  |  |  |  |  |
|  | **FRU** | **Total Area** | 15052 | 10793 | 14674 | 14546 |  |
|  |  | **Std. Error** | 215.7 | 228 | 371.5 | 277.1 |  |
|  |  | **95% Confidence Interval** | 14629 to 15475 | 10346 to 11239 | 13946 to 15402 | 14003 to 15089 |  |
|  |  |  |  |  |  |  |  |
|  | **TOTAL** | **Total Area** | 24281 | 18163 | 23900 | 23701 |  |
|  |  | **Std. Error** | 368.4 | 350.1 | 502.8 | 416.5 |  |
|  |  | **95% Confidence Interval** | 23559 to 25003 | 17477 to 18850 | 22914 to 24885 | 22885 to 24517 |  |
|  |  |  |  |  |  |  |  |
|  |  | **GLU/FRU** | 0.61 | 0.68 | 0.63 | 0.63 |  |
|  |  | **GLU area (% of WT)** | 1 | 0.80 | 1.00 | 0.99 |  |
|  |  | **FRU area (% of WT)** | 1 | 0.72 | 0.97 | 0.97 |  |
|  |  |  |  |  |  |  |  |
|  |  |  | **368 mg L^-1^ YAN** | | | |  |
|  |  |  | **AWRI 796** | **Tee9** | **Gid7(E726K)** | **Fba1(G135S)** |  |
|  | **GLU** | **Total Area** | 8893 | 6758 | 9105 | 9256 |  |
|  |  | **Std. Error** | 242.7 | 375.3 | 149.6 | 301.1 |  |
|  |  | **95% Confidence Interval** | 8417 to 9368 | 6022 to 7494 | 8812 to 9399 | 8666 to 9847 |  |
|  |  |  |  |  |  |  |  |
|  | **FRU** | **Total Area** | 14426 | 9692 | 15015 | 15115 |  |
|  |  | **Std. Error** | 350.7 | 462.2 | 311.2 | 350.7 |  |
|  |  | **95% Confidence Interval** | 13738 to 15114 | 8787 to 10598 | 14405 to 15625 | 14427 to 15802 |  |
|  |  |  |  |  |  |  |  |
|  | **TOTAL** | **Total Area** | 23307 | 16447 | 24114 | 24365 |  |
|  |  | **Std. Error** | 577.4 | 825.8 | 361.3 | 573.1 |  |
|  |  | **95% Confidence Interval** | 22176 to 24439 | 14828 to 18065 | 23406 to 24823 | 23242 to 25489 |  |
|  |  |  |  |  |  |  |  |
|  |  | **GLU/FRU** | 0.62 | 0.70 | 0.61 | 0.61 |  |
|  |  | **GLU area (% of WT)** | 1 | 0.76 | 1.02 | 1.04 |  |
|  |  | **FRU area (% of WT)** | 1 | 0.67 | 1.04 | 1.05 |  |
|  |  |  |  |  |  |  |  |
|  |  |  | **Semillon 96 mg L^-1^ YAN** | | | |  |
|  |  |  | **AWRI 796** | **Tee9** | **Gid7(E726K)** | **Fba1(G135S)** |  |
|  | **GLU** | **Total Area** | 5917 | 6130 | 6009 | 5836 |  |
|  |  | **Std. Error** | 76.29 | 43.90 | 34.83 | 40.56 |  |
|  |  | **95% Confidence Interval** | 5768 to 6067 | 6044 to 6216 | 5941 to 6077 | 5756 to 5915 |  |
|  |  |  |  |  |  |  |  |
|  | **FRU** | **Total Area** | 8209 | 8000 | 8054 | 8075 |  |
|  |  | **Std. Error** | 364.2 | 178.8 | 153.4 | 112.4 |  |
|  |  | **95% Confidence Interval** | 7495 to 8923 | 7650 to 8350 | 7753 to 8354 | 7855 to 8295 |  |
|  |  |  |  |  |  |  |  |
|  | **TOTAL** | **Total Area** | 14126 | 14130 | 14061 | 13911 |  |
|  |  | **Std. Error** | 431.9 | 173.3 | 154.8 | 96.39 |  |
|  |  | **95% Confidence Interval** | 13279 to 14972 | 13790 to 14470 | 13758 to 14365 | 13722 to 14100 |  |
|  |  |  |  |  |  |  |  |
|  |  | **GLU/FRU** | 0.72 | 0.77 | 0.75 | 0.72 |  |
|  |  | **GLU area (% of WT)** | 1 | 1.04 | 1.02 | 0.99 |  |
|  |  | **FRU area (% of WT)** | 1 | 0.97 | 0.98 | 0.98 |  |
|  |  |  |  |  |  |  |  |
|  | * Low N fermentation (83 mg L^-1^ YAN): Tee9 completed fermentation within 312 h, whilst the other 3 strains failed to finish at 384 h when the experiment was terminated. See Fig. 5. for sugar utilisation graphs. | | | | | |  |
|  |  |  |  |  |  |  |  |

**Supplementary Fig. S1**

**
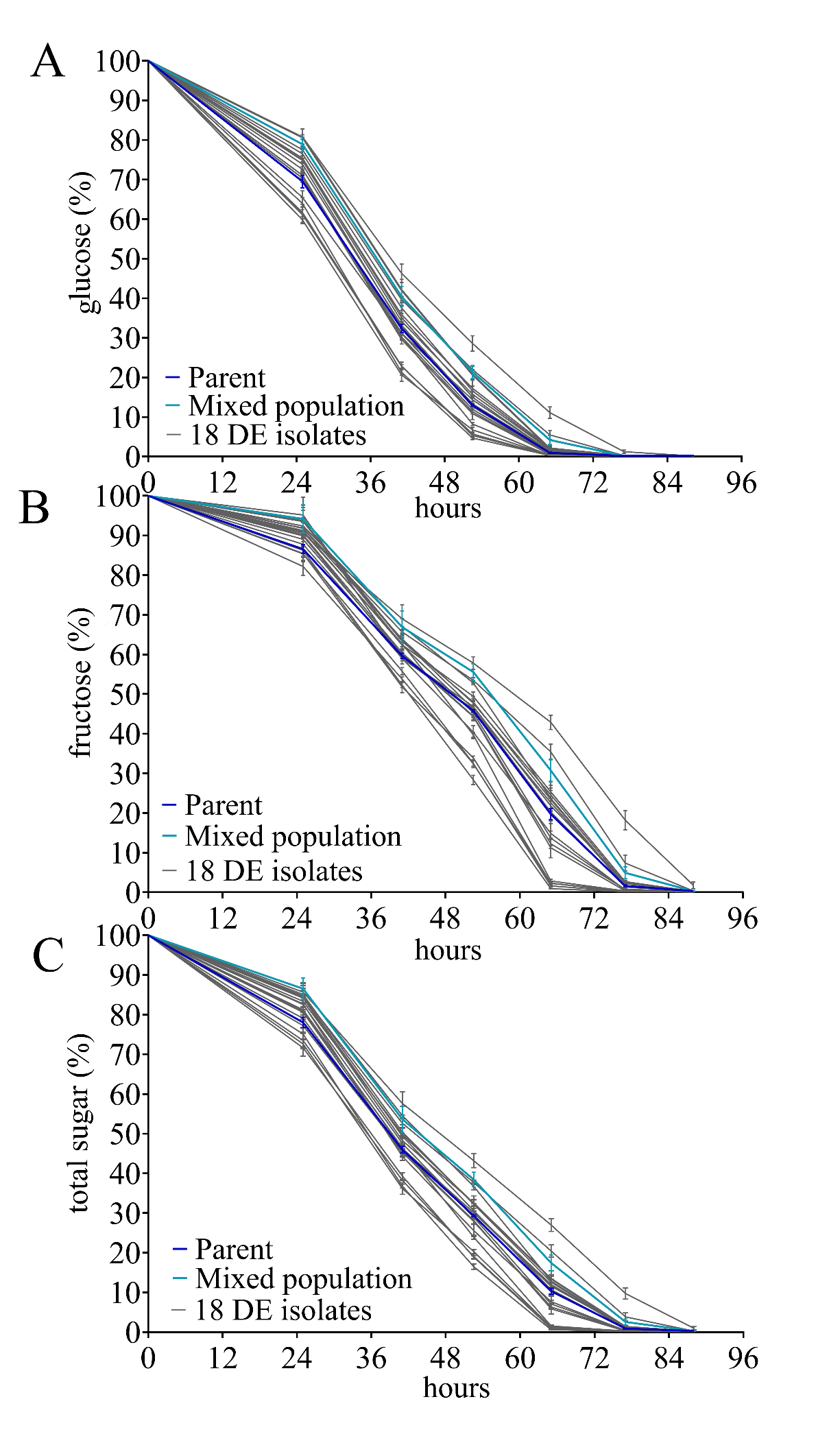
**

**Supplementary Fig. S2**

**
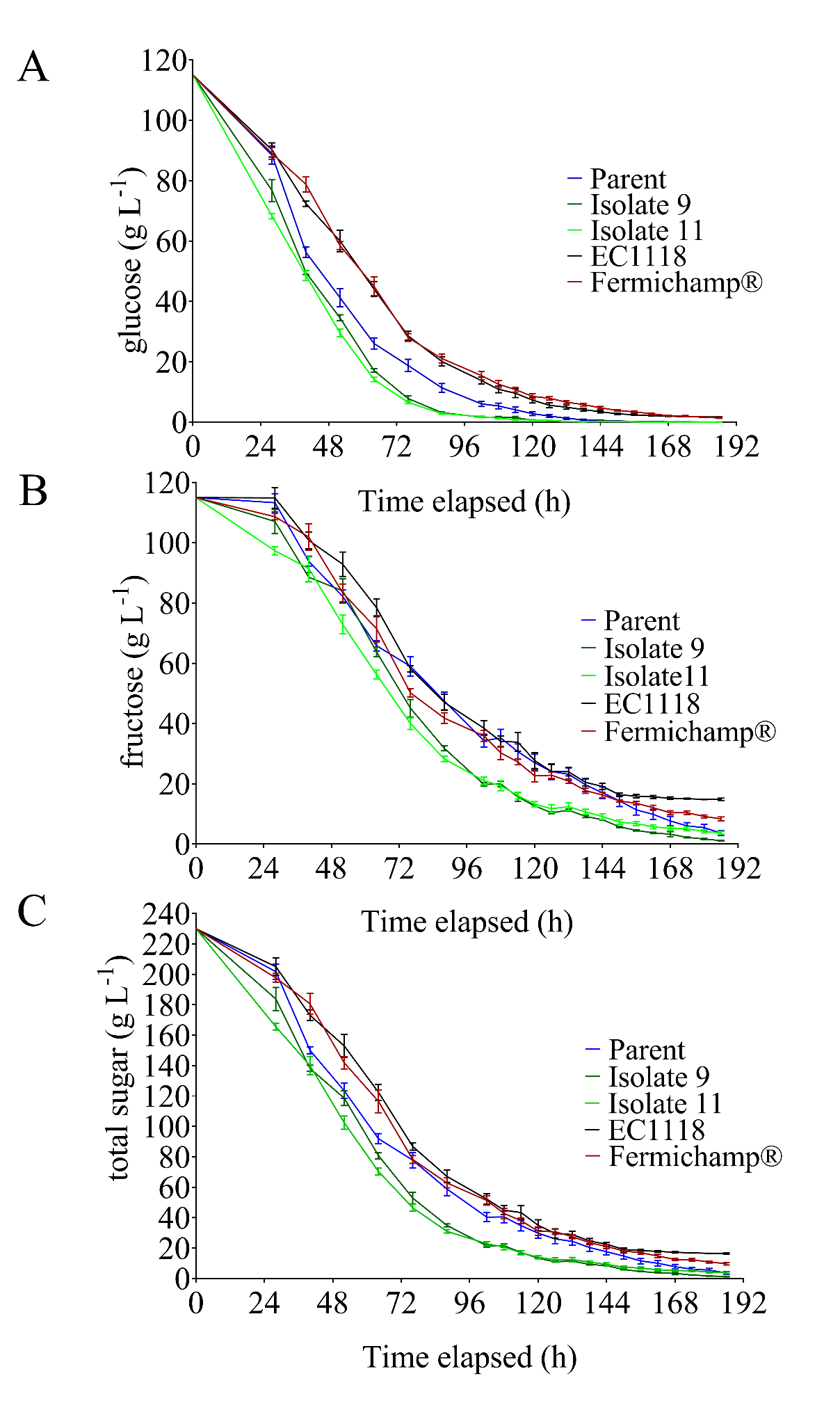
**

**Supplementary Fig. S3**


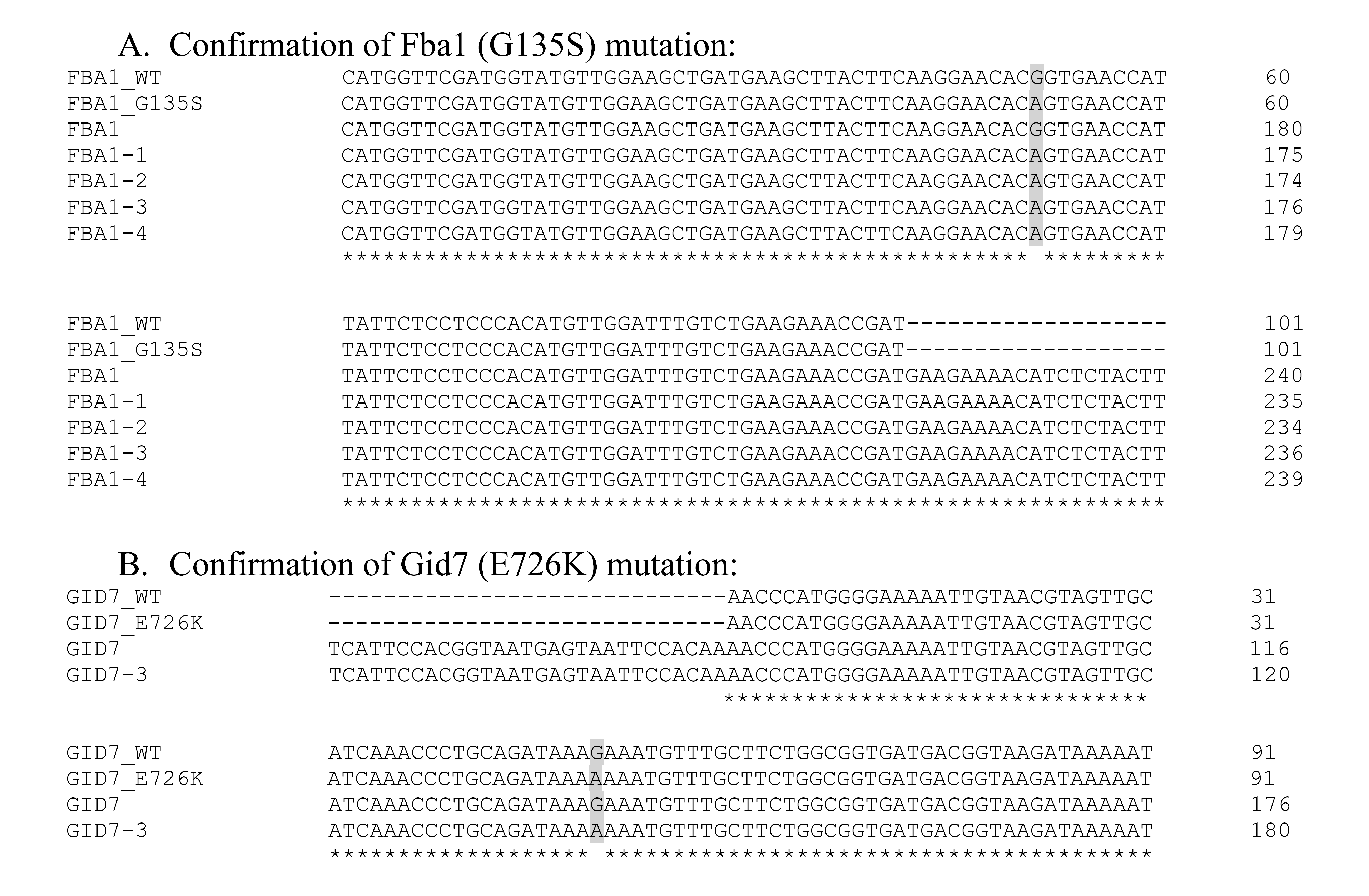


**Supplementary Fig. S4**

**
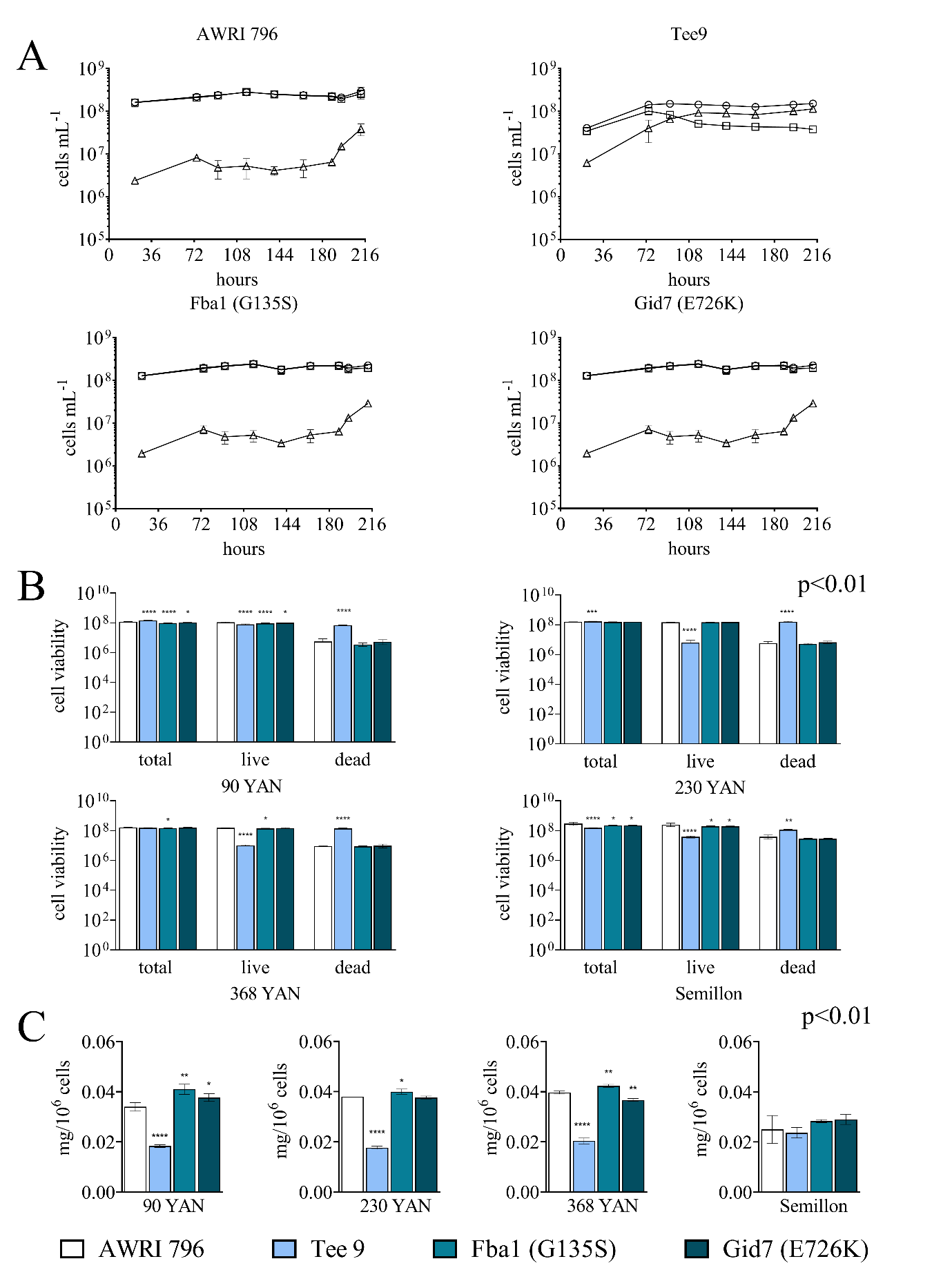
**

**Supplementary Fig. S5**

**
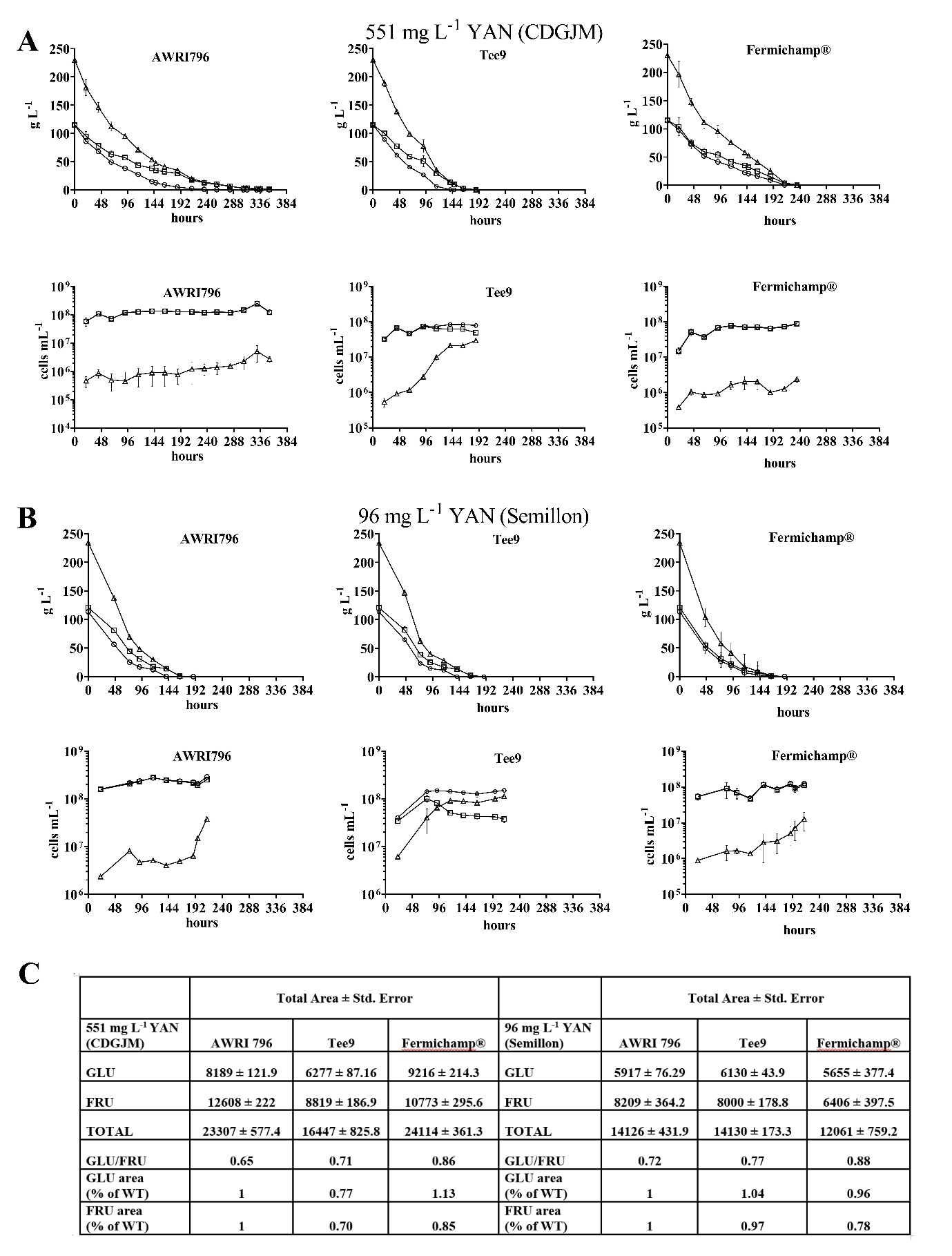
**

**Supplementary File S1 (Excel):** Raw data for Fig. 1. Area Under the Curve (AUC) values for glucose, fructose and total sugar utilisation are shown for the 54 isolates, parent and mixed population per 50-generational sample. GraphPad Prism was used to plot the data (Fig. 1) and statistical analysis (1-way ANOVA, Tukey’s multiple comparison tests).

**Supplementary File S2 (PDF):** Copy Number Variation analysis of chromosomes between AWRI 796 (published; SRR2967854) and the AWRI 796 isolate from this study (MP2 sequence)

**Supplementary File S3 (PDF)**

Allele Frequency Data between AWRI 796 (orange) and evolved strain, Tee 9 (blue). *De novo* homozygous SNPs are shown as red dots. Chr XIII region (grey bar) as possible result of mitotic recombination or gene conversion events.


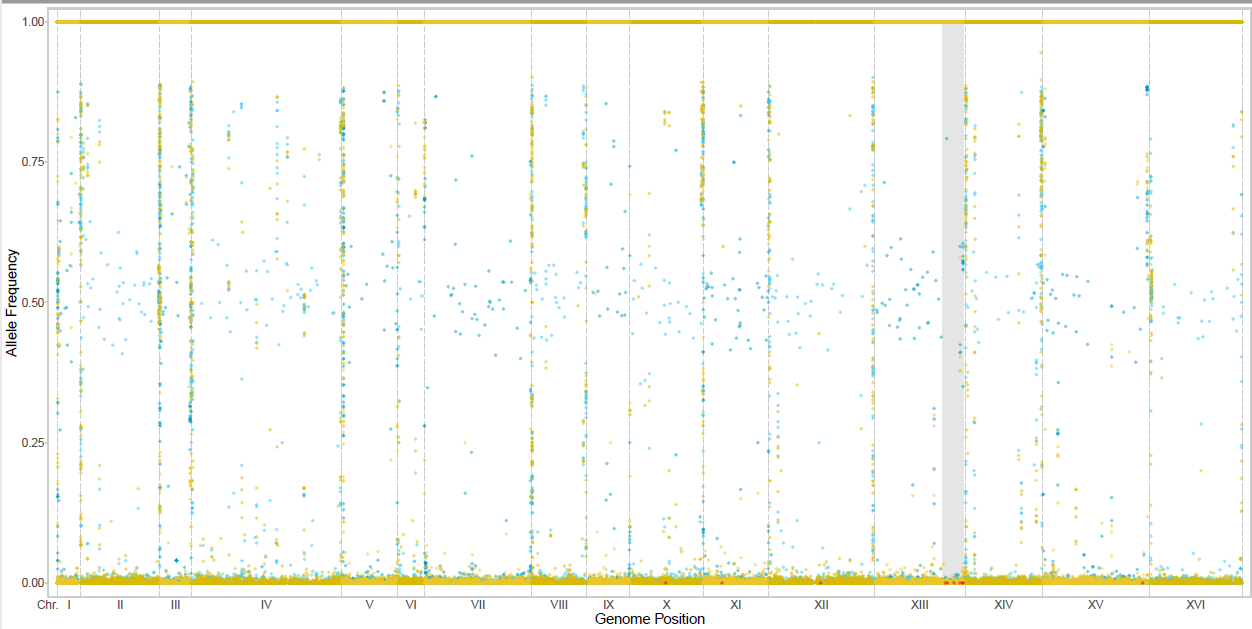


**Supplementary File S4 (PDF)**

Copy Number Variation analysis of chromosomes between AWRI 796 (orange) and evolved strain, Tee 9 (blue).


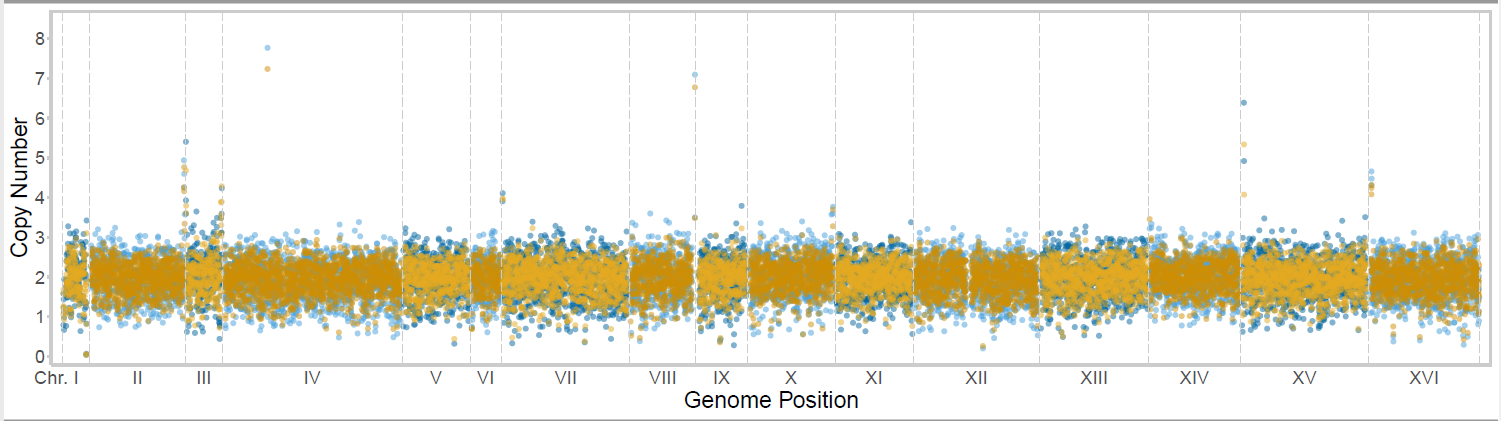


**Supplementary File S5:** Variant calling analysis identified 371 mutations in the evolved isolate Tee9 versus the parent (ancestral) strain AWRI 796 (Excel; see attached file; Tab 1). A total of 297 genes were mutated (Tab 2). 95 genes had non-coding mutations (Tab 3). 82 genes had synonymous mutations (Tab 4). 180 genes had non-synonymous SNPS (Tab 5) of which 98 were predicted to have an effect on protein structure (Tab 6-8). GO analysis of the 180 genes (Tabs 9-10) and 98 gene datasets (Tab 12-13) identified an enrichment of genes annotated to specific GO terms (Tabs 11 and 14).
